# Changes in Exosomal miRNA Composition in Thyroid Cancer Cells after Prolonged Exposure to Real Microgravity in Space

**DOI:** 10.3390/ijms222312841

**Published:** 2021-11-27

**Authors:** Petra M. Wise, Paolo Neviani, Stefan Riwaldt, Thomas J. Corydon, Markus Wehland, Markus Braun, Marcus Krüger, Manfred Infanger, Daniela Grimm

**Affiliations:** 1 The Saban Research Institute, Children’s Hospital Los Angeles, University of Southern California, 4650 Sunset Blvd., Los Angeles, CA 90027, USA; wisepetra@gmail.com (P.M.W.); pneviani@chla.usc.edu (P.N.); 2 Department of Microgravity and Translational Regenerative Medicine, Clinic for Plastic, Aesthetic and Hand Surgery, Otto von Guericke University, Universitätsplatz 2, 39106 Magdeburg, Germany; stefan.riwaldt@med.ovgu.de (S.R.); markus.wehland@med.ovgu.de (M.W.); marcus.krueger@med.ovgu.de (M.K.); manfred.infanger@med.ovgu.de (M.I.); 3 Department of Biomedicine, Aarhus University, Ole Worms Allé 4, 8000 Aarhus C, Denmark; corydon@biomed.au.dk; 4 Department of Ophthalmology, Aarhus University Hospital, 8200 Aarhus N, Denmark; 5 Research Group “Magdeburger Arbeitsgemeinschaft für Forschung unter Raumfahrt- und Schwerelosigkeitsbedingungen” (MARS), Otto von Guericke University, 39106 Magdeburg, Germany; 6 Deutsches Zentrum für Luft- und Raumfahrt (DLR), Raumfahrtagentur im DLR, Bonn-Oberkassel, 53227 Bonn, Germany; m.braun@dlr.de

**Keywords:** thyroid cancer, cell culture, exosomes, microgravity, spaceflight, miRNA

## Abstract

As much as space travel and exploration have been a goal since humankind looked up to the stars, the challenges coming with it are manifold and difficult to overcome. Therefore, researching the changes the human organism undergoes following exposure to weightlessness, on a cellular or a physiological level, is imperative to reach the goal of exploring space and new planets. Building on the results of our CellBox-1 experiment, where thyroid cancer cells were flown to the International Space Station, we are now taking advantage of the newest technological opportunities to gain more insight into the changes in cell–cell communication of these cells. Analyzing the exosomal microRNA composition after several days of microgravity might elucidate some of the proteomic changes we have reported earlier. An array scan of a total of 754 miRNA targets revealed more than 100 differentially expressed miRNAs in our samples, many of which have been implicated in thyroid disease in other studies.

## 1. Introduction

Space, the final frontier, is the ultimate goal for humans to explore. The fascination of the stars and the longing to reach them is as old as humankind itself. Through technological advancement combined with the age of information, space travel has become part of our reality. Multiple programs, manned or unmanned, have broadened our knowledge about space and the planets of our solar system; exploration of its planets and even commercial travel seems possible in the near future [1,2,3,4,5,6]. Still, research must find solutions to overcome multiple challenges to advance to these goals, not only on a technical level but also to prevent the rather dramatic effects the space environment wields over the human organism. The fact is that prolonged lack of exposure to gravitational forces can lead to numerous impairments in multiple physiological systems [7,8,9,10,11,12]. Therefore, researchers have started investigating the effects of weightlessness on the human body early in the history of spaceflight [13,14,15].

Even with the addition of several new enterprises in the field of interstellar travel, spaceflights are still rare and require extensive preparation and funding. Hence, most of the initial cell biological experiments are conducted on Earth under simulated microgravity (s-μ*g*) conditions. To adequately simulate weightlessness in a laboratory setting, disparate devices have been developed over the years, such as the random positioning machine (RPM) and clinostats [16,17,18,19,20].

The changes observed after cell incubation within these devices often predict cellular changes during spaceflight, but by direct comparison, s-μg and real microgravity (r-μ*g*) do not cause identical effects [21]. When our team was presented with the opportunity to conduct experiments under prolonged microgravity as part of a joint enterprise with the German Aerospace Center (Deutsches Zentrum für Luft- und Raumfahrt, DLR) (Bonn, Germany) and the Chinese Manned Space Engineering Office (CMSEO) (Beijing, PR China) [22] during the Shenzhou-8/SimBox Spaceflight mission in 2011, we took on the challenge. Both the planning and execution of cell culture experiments on the unmanned Shenzhou-8 spacecraft was a major undertaking. The cell culture equipment had to be developed to withstand the physical forces during the mission and had to be adapted to small, automated modules to enable fluid exchanges [23,24].

Extensive hardware tests prior to and during the Shenzhou-8 mission and the results and insights gained through the following analysis of the harvested samples provided us with the essential knowledge to equip a follow-up experiment under prolonged exposure to r-μ*g* conditions (r-μ*g*). Our team described changes of cancer cells from two to three-dimensional growth after exposure to simulated microgravity on the RPM before [24]. Moreover, genomic adaptations in pathways involved in the cytoskeletal composition were identified [25,26,27]. For the above-mentioned follow-up investigation, the human follicular thyroid cancer cell line FTC-133 was flown to the International Space Station (ISS) on the SpaceX CRS-3 cargo mission in newly designed, automated CellBox-1 modules, as described in more detail in Section 4.2. Incubation aboard the ISS proceeded for 12 days, following a pre-determined feeding and fixation schedule (flight module, FM). Concurrently, cells from the same passage were grown under the exact same conditions considering the hardware, temperature, and fluid exchange schedule in a laboratory on Earth (ground module, GM). The harvested cells and supernatant were analyzed to determine the growth behavior, adaptations in gene and protein expression, and protein interactions [26].

In an attempt to gain more knowledge on the underlying cellular changes of the differential protein expression following exposure to microgravity, we decided to re-analyze the CellBox-1 sample supernatants, this time regarding their exosome content. Exosomes are a group of extracellular vesicles (EVs), which have gained increasing interest and attention over the last decade. EVs are a family of lipid bilayer-delimited particles, which are naturally released into the extracellular environment by the majority of (if not all) cells and do not replicate [28,29,30]. Over the last decade, their high potential as biomarkers, as well as therapeutic vessels in clinical applications became apparent, due to their emerging roles in normal physiology, as well as various diseases. Diverse subtypes of EVs have been defined, but the three major types are categorized in exosomes, microvesicles, and apoptotic bodies. They are found in circulating body fluids, including blood, saliva, and urine [28,31,32,33,34]. EVs reflect their cell of origin with respect to their cargo, which consists of proteins, nucleic acids, lipids, metabolites, and even organelles from the parent cell. Initially thought to solely be a means of cellular waste disposal, more and more functional roles of EVs have been elucidated over the years, and concurrently, the interest in their implications and possible uses have led to the development of various methods of isolation and analysis [35,36,37,38]. Transfer of functional proteins and RNA, molecular recycling, creation of a metastatic niche for cancer and pathfinding through the environment, and quorum sensing are only a few [39,40,41,42,43,44,45,46]. EVs facilitate the horizontal transfer of information between different cells and are most likely the organism’s most important tool in cell–cell communication. With their potential to serve as biomarkers for a wide array of diseases in clinical diagnosis, the role of EVs in the tumor microenvironment has been studied extensively over the last decade. EVs are transferred from tumors to the surrounding extracellular environment and, thus, modulate the immune and therapy response, and promote matrix remodeling, as well as angiogenesis [47,48,49,50]. Conversely, the transfer of EVs from the tumor microenvironment to tumor cells enhances tumorigenesis by increasing tumor cell proliferation, resistance to chemotherapy, and migration [50,51,52,53,54,55]. As mentioned above, EVs can travel to distant areas of the organism, where they can be involved in the preparation of the pre-metastatic niche [39,40,41,42,43,44].

**Figure 1:**
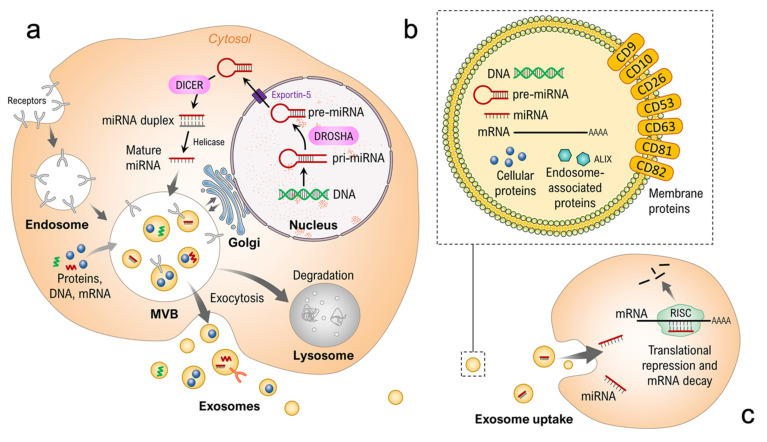
(**a**) Schematic overview of exosome biogenesis, cargo loading, and release and miRNA maturation. (**b**) Typical molecular contents of an exosome. (**c**) Exosomal uptake and post-translational protein regulation via miRNAs. Parts of the figure are drawn using pictures from the Servier Medical Art (https://smart.servier.com), licensed under a Creative Commons Attribution 3.0 Unported License (https://creativecommons.org/licenses/by/3.0).

Our groups’ interest is focused on exosomes, also known as small EVs, vesicles of endosomal origin defined by their size, biogenesis, and multiple well-defined markers. Exosomes range in size from roughly 30 to 150 nm and are further defined by the presence of specific transmembrane, extracellular, and cytosolic proteins, as well as the absence of some intracellular proteins present in other EVs. The International Society for Extracellular Vesicles (ISEV) has detailed the minimal experimental requirements for a definition of exosomes and other EVs [37,56]. Additionally, exosomes can be distinguished by their release mechanism to the extracellular environment via fusion of late endosomes/multivesicular bodies (MVBs) with the plasma membrane (Figure 1) [57,58]. The challenges of studying exosomes—their small size, low yields and/or co-precipitation of aggregated and bound proteins during isolation and enrichment—have been met by the research community with the development of new methods to characterize these promising particles. The most common methods of determining the size distribution and concentration of exosomes, along with the determination of cell origin and distinguishing between different EVs have been described in detail [59,60,61]. Previously, we have used a method based on the single-particle interferometric reflectance imaging sensor (SP-IRIS) (ExoView™, NanoView Biosciences, Boston, MA, USA), which permits multiplexed phenotyping and digital counting of various populations of individual exosomes captured on a microarray-based chip. This method allows the characterization of exosomes directly from human biofluids without the need for prior vesicle isolation or concentration and so grants a detailed analysis with a very small sample size [60,62,63].

Here, we are presenting the results of our continuative analysis of the exosomal composition of the CellBox-1 samples for integration with the entirety of our previous findings. To delve deeper into the regulatory changes of protein expression after prolonged exposure to r-μ*g*, we opted to analyze potential changes in the exosomal microRNA (miRNA) content. miRNAs are small, non-coding RNA molecules found in animals, plants and some viruses and are involved mainly in the post-transcriptional regulation of gene expression [64,65]. First discovered in 1993, their regulatory function was not elucidated until the early 2000s [66,67,68,69]. The majority of miRNA “genes” are located in intergenic regions or in antisense orientation in annotated genes [65,67,70]. They are transcribed as fairly long primary transcripts, the pri-miRNA, then transformed into the hairpin-shaped pre-miRNA by the RNase III Drosha [71,72]. After being transported into the cytoplasm, the pre-miRNA is cleaved to the mature miRNA by Dicer, a critical member of the RISC complex (RNA-induced silencing complex), with a length of 19–25 nucleotides [65,73,74,75] (Figure 1). miRNAs pair with mRNAs to direct post-transcriptional repression (mainly) by way of either direct translational repression, mRNA destabilization or both [66,76]. Another member of the RISC complex, Ago2, is activated by the miRNA/mRNA pairing and cleaves the mRNA [77]. The various molecular processes involved in miRNA-directed translational repression and mRNA destabilization, which include inhibition of translation initiation and poly(A) shortening, are reviewed in detail by Filipowicz et al. [78]. The number of defined miRNAs has passed 1200 and is rising continuously. With the large quantity of reported miRNAs in cancer research, our interest in this group of protein regulators was easily triggered, and our prospects were to find some common links between changes in the exosomal miRNA cargo and our previously reported genomic and proteomic cellular changes following extended exposure to r-μ*g*. Optimizing for source material, as well as the given hypotheses, is crucial, as the different protocols differ drastically in yield, purity, and functionality of the harvested EVs, as shown in comparative studies [35,79,80].

Likewise, the definition and description of EVs have varied greatly and proved overwhelming at times. As a result, the ISEV set the minimal required standard in defining the different vesicles to the report of amounts of several proteins (three or more) in at least a semi-quantitative manner in any EV preparation, including EV isolates obtained from body fluids or from secreting cells in vitro. The proteins described and characterized should be proteins expected to be present in the EVs of interest, especially transmembrane proteins and cytosolic proteins with membrane-binding capacity. In addition, the level of presence of proteins not expected to be enriched in EVs of endosomal origin should also be determined [56]. Transmembrane proteins expected to be present in exosomal preparations include, among others, tetraspanins (CD9, CD63, CD81), integrins, and growth factor receptors. The group of expected or enriched cytosolic proteins includes endosome or membrane-binding proteins (TSG101) and signal transduction or scaffolding proteins (syntenin). Intracellular proteins that are absent or under-represented in exosomes but present in other types of EVs are proteins found in the endoplasmic reticulum (ER) (calnexin) or the Golgi apparatus (GM130) [56].

## 2. Results

Exosomal miRNAs are instrumental in post-transcriptional regulation of protein expression and have earned recognition, particularly in tumorigenic pathways, including thyroid cancer [81,82,83,84]. With methods in place to engineer either cell-secreted or artificial exosomes loaded with a cargo of choice, as well as the possibility to target the recipient cell of choice precisely via surface protein presentation, the choice to scan our samples for differential miRNA expression was easy. The application of the TaqMan system is widely used and standardized, and therefore, offers numerous options for a thorough analysis. The EVs isolated from the CellBox-1 samples have been defined as exosomes in our previous publication [63]; hence we were able to use these EVs directly for any follow-up analyses.

### 2.1. Particle Concentration and Size Distribution Analysis by NanoSight

Following the exosome isolation via size exclusion chromatography (SEC) the concentration and size distribution of the harvested particles was evaluated by nanoparticle tracking analysis (NTA) using a NanoSight instrument. Since the EVs present in all samples were already characterized as exosomes previously, this single analysis is sufficient to validate the harvested particles. The two fractions per sample containing the desired particles were analyzed; for the GMs, the mean size ranged from 92.1 to 129.6 nm with an average of 107.3 nm. The mode, meaning the size of the majority of particles, ranged from 64 to 117.8 nm, averaging 86.9 nm. The FM mean particle size ranged from 94.3 to 114.9 nm with an average of 106.8 nm, and the mode ranged from 68.7 to 98.3 nm, averaging 83.8 nm.

**Figure 2:**
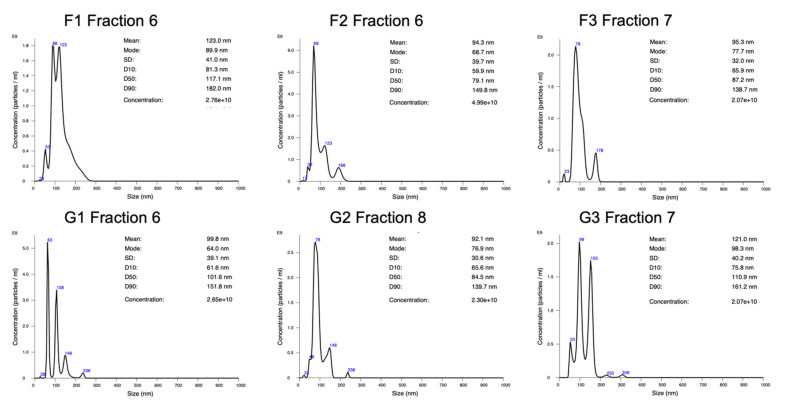
Size and concentration graphs of one of two fractions per sample. On display is the fraction with the higher particle concentration.

The concentration of the FM samples was determined at an average of 2.7 × 10^10^ particles/mL, ranging from 1.31 × 10^10^ to 4.99 × 10^10^ particles/mL; the GM samples averaged at a concentration of 2.0 × 10^10^ particles/mL in the range from 1.15 × 10^10^ to 2.65 × 10^10^ particles/mL. Figure 2 displays the size graphs for one fraction per sample, and the entire reports can be found in Supplementary Figure S1.

### 2.2. TaqMan™ Advanced miRNA Human Array Cards

The TaqMan™ Advanced miRNA Arrays offer a valuable first insight into any different expression patterns of any given samples, as the investigator is able to scan 754 targets in a streamlined and validated experimental setup. The human array cards have been developed to screen for miRNAs that have been reported as valuable markers in cancer and other relevant diseases. The analysis of total RNA cargo isolated from exosomes following our scan of one FM and GM CellBox-1 sample each disclosed a surprisingly large amount of differentially expressed miRNAs.

A total of 119 miRNAs were either elevated or decreased in the FM in comparison to the GM (Table 1). A total of 19 miRNAs displayed a higher relative quantification (RQ) value in FM, the percent increase ranging from 7% to 48%, with hsa-miR-553 leading the list. Hsa-miR-941 (138% increase), hsa-miR-1286 (132% increase), and hsa-miR-548d-5p (119% increase) also revealed more than double the expression compared to the GM. Expression of another four miRNAs was increased in the range of 94–81%: hsa-miR-154-3p (94%), hsa-miR-548g-3p (91%), hsa-miR-500a-3p (83%), and hsa-miR-583 (81%); the adjacent 11 miRNAs displayed expressional elevations between 52% and 7%.

**Table 1:** Differentially expressed microRNAs in FM compared to GM.

Target	RQ	% Change	Target	RQ	% Change
hsa-miR-553	5.476	448%	hsa-miR-28-5p	0.331	−67%
hsa-miR-941	2.382	138%	hsa-miR-1302	0.328	−67%
hsa-miR-1286	2.321	132%	hsa-miR-208b-3p	0.323	−68%
hsa-miR-548d-5p	2.191	119%	hsa-miR-30c-2-3p	0.320	−68%
hsa-miR-154-3p	1.944	94%	hsa-miR-367-3p	0.312	−69%
hsa-miR-548g-3p	1.913	91%	hsa-miR-1-3p	0.304	−70%
hsa-miR-500a-3p	1.827	83%	hsa-miR-452-5p	0.287	−71%
hsa-miR-583	1.814	81%	hsa-miR-197-3p	0.282	−72%
hsa-miR-221-5p	1.523	52%	hsa-miR-214-3p	0.276	−72%
hsa-miR-626	1.433	43%	hsa-miR-208a-3p	0.272	−73%
hsa-miR-16-5p	1.401	40%	hsa-miR-199a-5p	0.259	−74%
hsa-miR-1203	1.337	34%	hsa-miR-100-5p	0.259	−74%
hsa-miR-378a-3p	1.287	29%	hsa-miR-103a-3p	0.249	−75%
hsa-miR-452-3p	1.268	27%	hsa-miR-625-5p	0.247	−75%
hsa-miR-144-3p	1.180	18%	hsa-miR-376a-3p	0.246	−75%
hsa-miR-200a-5p	1.097	10%	hsa-miR-380-3p	0.242	−76%
hsa-miR-302d-3p	1.093	9%	hsa-miR-429	0.236	−76%
hsa-miR-646	1.084	8%	hsa-miR-125b-5p	0.235	−77%
hsa-miR-34b-3p	1.067	7%	hsa-miR-7-1-3p	0.231	−77%
hsa-miR-633	0.977	−2%	hsa-miR-128-3p	0.231	−77%
hsa-miR-1255a	0.952	−5%	hsa-miR-374b-5p	0.229	−77%
hsa-miR-524-3p	0.885	−11%	hsa-miR-323b-5p	0.229	−77%
hsa-miR-606	0.883	−12%	hsa-miR-653-5p	0.228	−77%
hsa-miR-1260a	0.875	−12%	hsa-miR-199a-3p_ hsa-miR-199b-3p	0.219	−78%
hsa-let-7b-3p	0.859	−14%			
hsa-miR-133b	0.842	−16%	hsa-miR-448	0.218	−78%
hsa-miR-645	0.841	−16%	hsa-miR-501-5p	0.217	−78%
hsa-miR-576-3p	0.839	−16%	hsa-miR-423-5p	0.197	−80%
hsa-miR-182-3p	0.823	−18%	hsa-miR-487a-3p	0.197	−80%
hsa-miR-548j-5p	0.816	−18%	hsa-miR-378a-5p	0.191	-81%
hsa-miR-27a-5p	0.806	−19%	hsa-miR-9-5p	0.191	−81%
hsa-miR-765	0.799	−20%	hsa-miR-374a-5p	0.191	−81%
hsa-miR-361-3p	0.787	−21%	hsa-miR-548a-3p	0.189	−81%
hsa-miR-628-3p	0.778	−22%	hsa-miR-325	0.186	−81%
hsa-miR-21-3p	0.761	−24%	hsa-miR-134-5p	0.184	−82%
hsa-miR-181c-3p	0.751	−25%	hsa-let-7d-5p	0.182	−82%
hsa-miR-770-5p	0.729	−27%	hsa-miR-31-5p	0.178	−82%
hsa-miR-34a-3p	0.725	−27%	hsa-miR-2110	0.170	−83%
hsa-miR-593-3p	0.660	−34%	hsa-miR-92a-3p	0.165	−83%
hsa-miR-548k	0.620	−38%	hsa-miR-148b-3p	0.164	−84%
hsa-miR-552-3p	0.612	−39%	hsa-miR-384	0.157	−84%
hsa-miR-562	0.594	−41%	hsa-miR-502-3p	0.155	−85%
hsa-miR-10b-3p	0.562	−44%	hsa-miR-148a-3p	0.149	−85%
hsa-miR-548n	0.544	−46%	hsa-miR-342-5p	0.145	−86%
hsa-miR-564	0.541	−46%	hsa-miR-200a-3p	0.138	−86%
hsa-miR-551a	0.521	−48%	hsa-miR-490-3p	0.138	−86%
hsa-miR-196a-5p	0.511	−49%	hsa-miR-200c-3p	0.136	−86%
hsa-miR-516b-5p	0.480	−52%	hsa-miR-744-5p	0.135	−87%
hsa-miR-190a-5p	0.467	−53%	hsa-miR-25-3p	0.122	−88%
hsa-miR-133a-3p	0.449	−55%	hsa-miR-450b-3p	0.118	−88%
hsa-miR-181b-5p	0.444	−56%	hsa-miR-27b-3p	0.108	−89%
hsa-miR-153-3p	0.417	−58%	hsa-miR-485-5p	0.100	−90%
hsa-miR-101-3p	0.417	−58%	hsa-miR-483-5p	0.098	−90%
hsa-miR-18a-3p	0.411	−59%	hsa-miR-518f-3p	0.097	−90%
hsa-miR-548e-3p	0.402	−60%	hsa-miR-525-3p	0.091	−91%
hsa-miR-147a	0.397	−60%	hsa-miR-567	0.089	−91%
hsa-miR-338-3p	0.368	−63%	hsa-miR-324-5p	0.084	−92%
hsa-miR-660-5p	0.346	−65%	hsa-miR-339-3p	0.070	−93%
hsa-miR-518c-3p	0.335	−66%	hsa-miR-518d-3p	0.069	−93%
hsa-miR-137	0.332	−67%	hsa-miR-501-3p	0.066	−93%

Of the 100 scanned miRNA targets with decreased RQ values, the percentage ranged from 2% to 93%; the detailed list can be found in Table 1. Briefly, 12 miRNAs displayed expression decreases below 20% (2–19%), and 16 ranged between 20% and 49%. The largest group could be found in the range between 50% and 80%, namely 39 of all scanned targets. A total of 24 miRNAs could be found grouped together with an expression decrease between 81% and 89%. Lastly, the nine remaining targets with the largest decrease in expression were the following: hsa-miR-485-5p, hsa-miR-483-5p, hsa-miR-518f-3p (all −90%), hsa-miR-525-3p, hsa-miR-567 (both −91%), hsa-miR-324-5p (−92%), and hsa-miR-339-3p, hsa-miR-518d-3p, hsa-miR-501-3p (all −93%).

**Figure 3:**
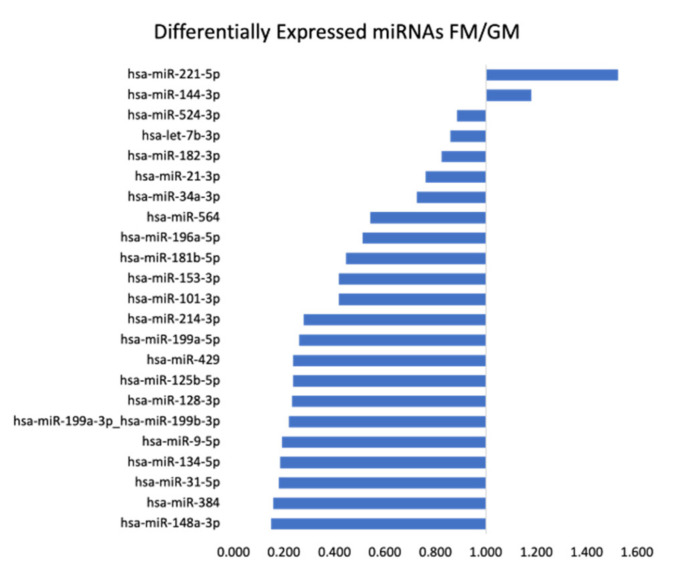
Differentially expressed miRNAs comparing FM with GM with TaqMan™ Advanced miRNA with the relevant impact in thyroid cancer.

Following another thorough in silico investigation, we discovered that a total of 23 of these differentially expressed miRNAs have been described in the pathogenesis of various thyroid cancers previously [84]. Hence, we decided to focus on these oncomiRs and tumor suppressors for further verification. Only two of the overexpressed miRNAs in the FM compared to the GM were found in this group, namely hsa-miR-221-5p (52% increase) and hsa-miR-144-3p (18% increase). The remaining 21 miRNAs were downregulated in varying degrees, starting from an 11% decrease up to an 85% decrease in the case of hsa-miR-148a-3p, as displayed in detail in Figure 3.

### 2.3. TaqMan™ Advanced miRNA Single Target Assays

Even though an application as a biomarker usually comes to mind first and would be easiest to utilize, two facts discouraged us from choosing this route: (a) the number of overexpressed miRNAs in our FM sample was distinctly lower compared to the GM, and (b) only two of these were reported as oncomiRs in thyroid cancer. Namely, hsa-miR-221-5p was upregulated most, but the 3p-strand is the one described in papillary thyroid carcinoma (PTC) previously [81,82,84,85,86]. The second oncomiR overexpressed in FM, hsa-miR-144-3p, is the lower expressed strand of the hairpin; hence most of the reports regarding hsa-miR-144 in PTC and MTC (medullary thyroid carcinoma) were evaluating the 5p strand [83,87]. Therefore, we decided to focus on the more intriguing tumor suppressors.

Three of these tumor-suppressor miRNAs were chosen to be validated using TaqMan™ Advanced Single Target assays: hsa-miR-429 (decreased by 76%), hsa-miR-128-3p (77% decrease), and hsa-miR-199a-5p (downregulated 78%). All three have been described in thyroid tumors previously [88,89,90,91].

#### 2.3.1. hsa-miR-429

**Figure 4:**
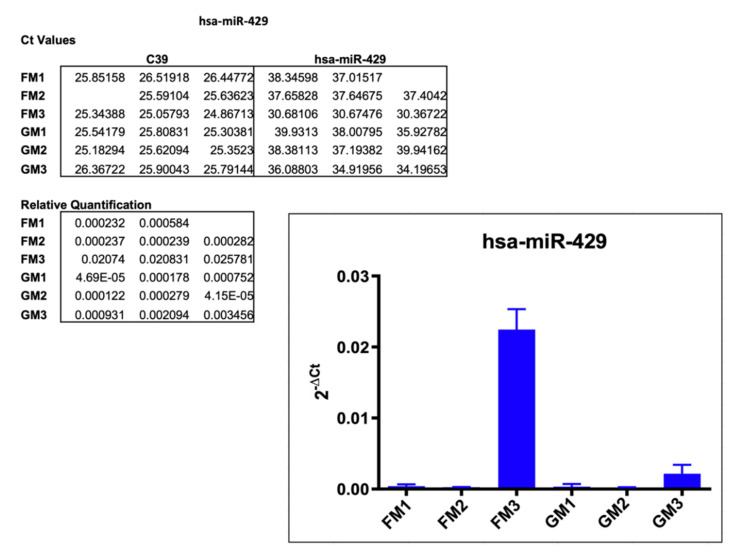
Results of the single assay analysis of hsa-miR-429, including Ct values, relative quantification, and the graphical representation of the results. Empty spots in the listed Ct values did not produce any measurable amplification.

In the initial TaqMan™ Advanced miRNA Array scan, the hsa-miR-429 expression was decreased by 76% in FM compared to GM. In a follow-up single assay, all FM and GM samples were analyzed, resulting in an increased expression across the board of varying degrees. The C_t_ values in all triplicates of FM1 and FM2 were at around 37 to 38 and averaged 38.23 in GM1 and GM1. Normalization with C39 led to relative quantification values of 0.0004 and 0.00025 (FM1, FM2) vs. 0.00032 and 0.00014. FM3 though displayed a much more dominant increase in miR-429 expression, at C_t_ values of 30 to 31 (FM3) compared to 35.1 (GM1) with a resulting relative quantification of 0.022 (FM3) vs. 0.0021 (GM3) (Figure 4).

#### 2.3.2. hsa-miR-128-3p

**Figure 5:**
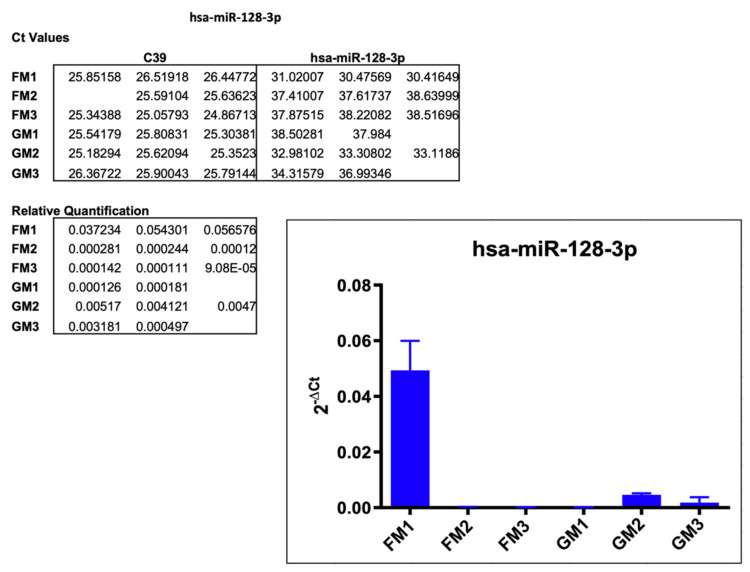
Results of the single assay analysis of hsa-miR-128-3p, including Ct values, relative quantification, and the graphical representation of the results. Empty spots in the listed Ct values did not produce any measurable amplification.

Based on a 77% decrease in miR-128-3p expression in the array scan, the results of the single assay analysis delivered mixed results. As depicted in Figure 5, FM2 and FM3 supported this trend amplifying at C_t_ values between 37.4 and 38.2, compared to the GM values ranging at an average of 34.1, leading to a relative quantification of 0.0002 and 0.0001 vs. 0.0046 and 0.0018 on average. FM1, on the other hand, showed an increase in expression at C_t_ values from 30.4 to 31 vs. GM at values at around 38, with the respective average in relative quantification of 0.049 (FM1) and 0.00015 (GM1). These inconsistent results may have various underlying reasons, as the positioning of the modules on the racks may have subjected these samples to varying extents of different in-flight stressors, such as space radiation or acceleration forces, as described in the discussion.

#### 2.3.3. hsa-miR-199a-3p_hsa-miR199b-3p

**Figure 6:**
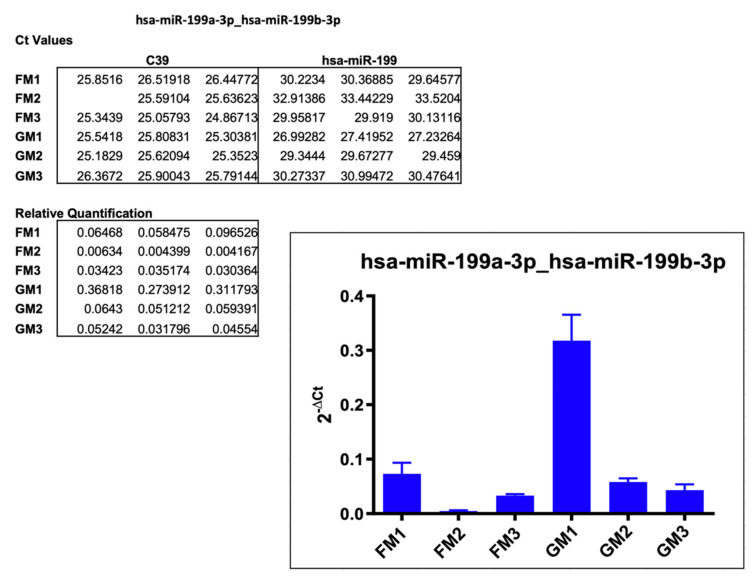
Results of the single assay analysis of hsa-miR-199a-3p_hsa-miR-199b-3p, including Ct values, relative quantification, and the graphical representation of the results. Empty spots in the listed Ct values did not produce any measurable amplification.

The last miR chosen for validation via single assay was hsa-miR-199a-3p_hsa-199b-3p. The expression decrease in the array scan was 78% in FM vs. GM, and this trend could be validated across all three sample sets, as shown in Figure 6. With average relative quantification values of 0.07 vs. 0.31 (FM1 vs. GM1), 0.004 vs. 0.05 (FM2 vs. GM2), and 0.03 vs. 0.04 (FM3 vs. GM3), all exosomes isolated from FM contained a lower amount of miR-199. The underlying C_t_ values were in the range from 29.64 to 33.52, with an average of 31.12 (FMs) and 26.99 to 30.99 with an average of 29.09 (GMs).

## 3. Discussion

Ever since the first manned space missions, the experienced effects of microgravity on the human organism have been a focal point of space research. Overcoming these extensive changes and adaptations, which span from gene expression alterations and cellular modifications to physiological adjustments, is mandatory to successfully continue the quest of space exploration and travel. Extensive studies on the occurrent physiological adaptations to the cardiovascular, musculoskeletal, and sensorimotor systems and the implications of varying durations of spaceflight were undertaken, and processes to prepare and recondition space travelers were developed [92,93,94]. Still, despite all efforts and scientific advances, many questions remain unanswered and are challenging space explorers, so the search for further insights continues.

In recent years, our group has studied multiple cell culture systems regarding their genomic and proteomic adaptations resulting from exposure to various conditions of weightlessness [25,26,95,96]. One of these systems we have explored is thyroid cancer, an endocrine tumor of continuously increasing incidence over the last decades [97,98]. To expand our knowledge further and to better understand the underlying cellular pathways leading to the demonstrated changes, we decided to re-evaluate the CellBox-1 samples in regard to their EV content and composition. EVs have taken a prominent place in the rankings of intercellular communication and post-transcriptional proteomic regulation over the last decade, and their significance as disease biomarkers and potential pharmaceuticals should not be underestimated [31,99,100,101,102]. Recently, we described the changes in exosomal secretion and populations under prolonged exposure to microgravity in the human follicular thyroid cancer cell line FTC-133, which our group has studied in various conditions of weightlessness before [24,63,103]. In these preliminary experiments on the exosomal secretion, we determined the number of secreted vesicles, their size distribution, and their population regarding the tetraspanin surface expression to evaluate the influence of microgravity on changes in the cellular cross-talk [63].

The impact of non-coding RNAs on regulatory processes in cells has been found to be more and more prominent, particularly in tumor networks [104,105,106,107]. Small non-coding RNAs, or miRNAs, have been described as highly adaptable cargo in tumor-associated exosomes, secreted from not only tumor cells but also from tumor-associated cells or immune cells. Given the ever-growing, vast pool of knowledge in this field, as well as the availability of new methods to re-analyze the samples from our prior experiments, miRNAs suggested themselves as the next logical targets. To get an initial overview of any given changes in our flight samples compared to the ground controls, we set up an initial screen over a total of 754 miRNA targets using the TaqMan™ array methodology. Surprisingly, we found almost 120 miRNAs differentially expressed in our samples. To narrow the selection of targets of interest for further validation, we analyzed the list filtering for miRNAs previously reported in thyroid cancer. This resulted in a pool of 23 miRNAs, 2 of them overexpressed in FM compared to GM; the remaining 21 were downregulated (Figure 3). From this group, we selected three miRNAs to be validated in all CellBox-1 samples via TaqMan™ single assays.

Before discussing these miRNAs, we would like to direct the reader’s attention to one miRNA, which was neither part of the list of previously described in thyroid cancer targets and hence, was not further evaluated in this study. We would like to include and point out hsa-miR-553 in our discussion, particularly because of its extraordinary high expression difference in the array screening. As recorded in Table 1, the RQ value of the FM was 5.476, which corresponds to the quite astonishing increase of 448% compared to our calibrator, GM. hsa-miR-553 was first specified by Cummins et al. [108] in 2006 as part of the colorectal miRNAome, which was determined via an extensive screening of non-coding RNAs tags in human colorectal cells. Ever since this miRNA has been reported on only one more time in relation to a malignant disease: Liang et al. revealed that miR-553 promoted proliferation, metastasis, and tumor-associated macrophage (TAM) resistance in MCF-7 and MCF-7/TAMR breast cancer cells; and high expression levels of miR-599 was correlated with poor prognosis. The circular RNA circBMPR2 served as a miR-553 sponge and decreased the abundance of miR-553 in the cytoplasm, according to the authors. Vice versa, miR-553 overexpression could partly reverse the effects of circBMPR2 on breast cancer cells. Moreover, circBMPR2 and miR-553 could regulate the expression of each other, forming a negative-feedback loop [109]. Therefore, despite the impressive increase of hsa-miR-553, we decided to not further investigate this miRNA at this time but rather shift our focus on several of the tumor suppressors described in thyroid cancers, as this might offer valuable insights to pathways we have described in FTC-133 prior, and which could be targeted in an effort to reduce tumorigenicity or even point towards possible therapeutic approaches.

As mentioned previously, the oncomiRs are a promising and logical target in tumor and exosome research. Significantly in EVs, upregulated miRNAs can be easily measured via liquid biopsies, and with possible specificity to any particular cancer, can easily serve as biomarkers and for disease staging. However, in view of the possibilities that exosomes offer regarding cargo loading and targeted delivery, tumor suppressors are a much more fascinating topic to explore. The prospects in developing and using tumor suppressor-laden exosomes to specifically target tumor cells and to circumvent their adaptations to the tumor environment and the organism’s immune response are worth the efforts. Hence, we were focusing on three miRNAs previously distinguished as tumor suppressors in thyroid cancer and additionally downregulated in our FM sample. This downregulation would be consistent with an increase in malignancy as a result of the extended exposure to microgravity, which supports our previous results within this cell line and other cell systems we have worked with [24,26,27,103,110].

### 3.1. hsa-miR-429

hsa-miR-429 is a member of the miR-200 family, along with miR-200a, miR-200b, miR-200c, and miR-141, and its genetic location can be found on chromosome 1 [111]. The evidence points to an involvement of miR-429 dysregulation in EMT, progression, development, invasion, metastasis, apoptosis, and drug resistance [112,113,114]. Similar to many other miRNAs, miR-429 has been characterized in multiple different tissues and types of cancers and has been portrayed as a relevant contributor to tumorigenesis as either a tumor suppressor or promoter in very tumor-specific patterns [88,115,116,117]. Aside from thyroid cancer, tumor-suppressive properties have been reported for miR-429 in breast cancer, cervical cancer, gastric cancer, glioblastoma, and osteosarcoma, to only name a few. An association of miR-429 in tumor progression, proliferation, migration, invasion, and metastasis has been discussed in this regard, as well as the induction of apoptosis, cell cycle arrest, and drug resistance in instances [118]. Contrastingly, miR-429 has displayed oncogenic characteristics in lung cancer, prostate cancer, and endometrial carcinoma. Here also, the miR is correlated with tumor development, progression, proliferation, and drug resistance. In certain tumors, namely colorectal cancer, bladder cancer, and hepatocellular carcinomas, miR-429 still has a quite paradoxical role—it has been reported as up and downregulated in the same tissues with the concurrent results. The type of involvement is consistent with the previously described conditions [118].

In thyroid cancer, miR-429 was evaluated in clinical samples and cell lines alike. The study authors found miR-429 significantly decreased studying 59 pairs of primary tumors and nontumorous control tissues, and transfection of miR-429 in thyroid cancer cell lines substantially inhibited cell proliferation, migration, and invasion ability. Moreover, miR-429 upregulation-induced apoptosis in several cell lines. Zinc finger E-box-binding homeobox 1 (ZEB1) was identified as a direct target of miR-429, and miR-429 transfection could inhibit ZEB1 by direct binding to its 3′-untranslated region (3′-UTR). ZEB1 is a well-known player in cancer, driving EMT, metastasis, and therapy resistance in many human cancers [119,120,121]. ZEB1 owes its pivotal role to the downregulation of E-cadherin and the regulation of further proteins involved in tumor progression, such as Crumbs3, HUGL2, and PATJ (Pals1-associated tight junction). Therapy resistance is not only limited to chemotherapy resistance but is also reported to influence radioresistance [120,122]. Further, ZEB1 reportedly regulates the development of an inflammatory phenotype and the activation of macrophages towards TAMs [122,123]. These data clearly suggest a role as a tumor suppressor in this disease pattern; our recent results substantiate these findings. We found that hsa-miR-429 was downregulated 76% in FM compared to GM in our initial scan, but we could not solidly validate these results with the following single target assay.

### 3.2. hsa-miR-128-3p

Cleaved from the stem-loop hsa-miR-128-2, miR-128-3p is the main mature strand with reports in more than 150 published experiments. It has been described—besides cancer—in various diseases physiological processes, such as rheumatoid arthritis, multiple sclerosis, atrial fibrosis, and vascular disease, response to sepsis and acute kidney injury, osteogenesis and bone healing, to name a few [124,125,126,127,128,129]. Mets et al. reported miR-128-3p as a candidate for an oncomiR in T-cell acute lymphoblastic leukemia as it downregulates PHF6, which plays an important tumor-suppressive role in this disorder. Other than that, miR-128-3p is mainly described in cancer as a tumor suppressor by inhibiting most processes during tumor development and progression, and the examples are manifold. Downregulating this miR accelerates progression in prostate, ovarian, and breast cancer, and in cervical cancer proliferation, migration, and invasion increases with diminishing miR-129-3p levels [130,131,132,133,134]. Additionally, miR-128-3p has been shown to increase chemosensitivity in colorectal cancer after transmission via exosomes or nanoparticles, making it a prime target in the attempt to overcome therapy resistance.

In a study examining papillary thyroid cancer (PTC) and follicular thyroid carcinoma (FTC) tissues and various thyroid carcinoma cell lines, miR-128 expression was markedly downregulated. Functionally over-expressing the miR markedly inhibited cancer cell migration and invasion, and these processes were reversible by silencing miR-128 expressions in thyroid tumor cells, leading to reduced apoptosis, enhanced proliferation, and metastasis. These results were supported by an in vivo xenograft tumor model where the overexpression of miR-128 reduced the tumor growth rate and tumor weight [89]. Interestingly, miR-128 also seems to target ZEB1 in a tumor-suppressive manner. The circular RNA circ-ABCB10 is a strong promoter of tumorigenesis in breast and cervical cancer. Circ-ABCB10 acts as a sponge for miR-128, which in turn results in an increase in its direct target ZEB1, leading to augmented cell proliferation, migration, invasion, and inhibition of apoptosis in cervical cancer [135]. In esophageal squamous-cell cancer (ESCC), ZEB1 is a crucial mediator of epithelial-mesenchymal transition and induces malignant progression. In their study, Zhao et al. found that miR-128-3p was downregulated in ESCC tissues and cells and downregulated expression of miR-128-3p was testified to be associated with poor prognosis of ESCC patients. In vitro and in vivo, the authors demonstrated that miR-128-3p could suppress cell migration, invasion, and metastasis [136]. As with miR-429, our array scan shows a downregulation of miR-128-3p, and we were able to verify this in two samples during the single assay analysis. This is in accordance with a rise in tumorigenicity in the FM samples in FTC-133 and a development towards a more aggressive phenotype, which has been observed in previous studies.

### 3.3. hsa-miR-199

The hsa-miR-199 family consists of three stem-loops, which all are cleaved into two mature miRNAs: hsa-miR-199b, hsa-miR-199a-1, and hsa-miR-199a-2. As listed in Table 1, in our initial screening array, we found three mature miRs of this family downregulated, miR-199a-3p, miR-199b-3p, and miR-199a-5p, and all three of these have been previously described in thyroid cancers as tumor suppressors [84]. The selection to validate miR-199a-5p was made due to mechanical advantages. As all previously-discussed miRs, hsa-miR-199a-5p has been reported as an oncomiR in a few malignancies, but mostly the mature l miRs of the entire family has been associated with tumor suppression. Our chosen target inhibits proliferation, migration, and invasion in colorectal cancer by negatively regulating integrin α3β1 (ITGA3), which has been linked to intercellular communication and serves an important role in the signaling among cells and the extracellular matrix. In addition, it was suggested that miR-199a-5p overexpression suppresses the EMT of colorectal cancer cells, whereas the overexpression of ITGA3 restores this effect [137]. The same holds true by looking at another miR199a-5p direct target, ROCK1 (Rho-associated coiled coil-containing protein kinase 1), Zhu and colleagues demonstrated in their study that microRNA-199a-5p was significantly downregulated in colorectal cancer cell lines and tissue samples and was associated with a poor prognostic phenotype. MicroRNA-199a-5p suppressed colorectal cancer cell proliferation, migration, and invasion and induced cell apoptosis [138]. Contrastingly, in cervical cancer, miR-199a-5p promotes the same aspects—proliferation, migration, and EMT—via direct targeting of protein inhibitors of activated signal transducer and activators of transcription 3 (PIAS3). PIAS3 is a regulator protein of other key transcription factors, including MITF, NFκB, SMAD, and estrogen receptors [139,140].

Looking at thyroid tumors, the miR-199 family has been reported as aiding in the suppression of cell proliferation, invasion, metastasis, and enhancing apoptosis. The ectopic expression level of miR-199b-5p in papillary thyroid carcinoma cell B-CPAP could inhibit growth, migration, and invasion, as well as epithelial-mesenchymal transition (EMT) and decreased cell metastasis in vivo, and alongside it improved the sensitivity of thyroid carcinoma cells to chemotherapy. Silencing miR-199b-5p caused contradictory outcomes [84,141]. miR-199a-3p also seems to play an important role in tumor inhibition. A link between miR-199a-3p expression in thyroid tissues and clinicopathologic features was evaluated by Liu and colleagues, as well as its potential usefulness in prediction for invasion and metastasis of PTC [84,90]. Lastly, miR-199a-5p has been depicted as an inhibitor of progression, cell proliferation, migration, and invasion and a promoter of apoptosis in PTC [91,142]. In addition, it was identified as a potential diagnostic biomarker in this disease [143]. Turning our attention to follicular thyroid carcinoma, the tumor type corresponding with our CellBox-1 samples, mir-199a-5p, has been reported to act via suppressing connective tissue growth factor (CTGF). Through comparison of clinical FTC samples and physiological controls, an under-expression of miR-199a-5p in FTC tissue samples combined with overexpression of CTGF was identified, proving a negatively correlated relationship between CTGF and miR-199a-5p [144]. In this miR, our study results clearly demonstrate a reduction in expression across the array scan, as well as the individual single assay analysis, which explains a development towards a more tumorigenic state of the FM samples in deactivating the tumor-suppressive potency of this group of miRs. With a decrease of miR-199 expression, the aggressiveness and invasiveness of the tumor cells should increase, and previous studies have pointed to such a development after exposure to microgravity conditions due to the various stressors the cells are subjected to [145].

To tie in this miR with the two previously discussed, miR-199a-5p also targets ZEB1 in multiple malignancies. It confers a tumor-suppressive role in triple-negative breast cancer (TNBC), in retarding proliferation, migration, invasion, and stem cell-like characteristics. The authors demonstrated modulation of EMT genes and stem cancer markers and suspect that miR-199a-5p may be involved in chemotherapy sensitivity in TNBC [146]. Further, through targeting ZEB1, miR-199a-5p inhibits EMT of ovarian ectopic endometrial stromal cells via the PI3K/Akt/mTOR signal pathway and in human renal tubular epithelial cells [147,148].

All in all, our studies show solid support for the hypothesis that these miRs have a role in reducing tumorigenesis in thyroid cancer. Their decrease in FM samples, as shown in the scan and the majority of single assays, match the previously-described intensification of oncogenic properties in thyroid cancer cells after subjection to s- and r-μ*g*. The variance in some samples could be due to the exact positioning of the FMs during the spaceflight, which would subject the cells to different intensities of various other stressors besides microgravity, such as space irradiation, to just name one. This study shows once more the difficulties one is facing in space research since every change in the external conditions may lead to unexpected or never-before observed adaptations.

It has been amply demonstrated that post-transcriptional protein regulation through miRNAs is manifold but also very specific in regard to the particular tissues and environments. However, with this in mind, the possibilities of finding common denominators within the vast number of targets are always abounded. As shown in this publication, microRNA research is a great starting point to find new angles and viewpoints to an otherwise well-known picture. This holds true for the additional angle of microgravity research. Our group has demonstrated the benefit of utilizing real or s-μ*g* in solving some of the riddles of tumor biology despite the challenges. With the aid of recently-developed methods and technical advances in exosome and miRNA research, we are opening another door to a better understanding of cancer and to the development of new therapeutic routes to battle this devastating disease.

## 4. Materials and Methods

### 4.1. Cell Cultures

Human follicular thyroid cancer cells (FTC-133), purchased from the Health Protection Agency Culture Collections (HPACC, Salisbury, UK), were cultured in RPMI-1640 (Invitrogen, Eggenstein, Germany), supplemented with 10% fetal calf serum (FCS, Biochrom, Berlin, Germany), penicillin (100 U/mL; Merck Millipore, Burlington, MA, USA), and streptomycin (100 µg/mL; Merck Millipore) at 37 °C and 5% CO_2_, as described previously [26]. Prior to the start of the experiment, 10^6^ cells were loaded into an automated cell culture system, the FM hardware [24], or alternatively seeded in T-25 culture flasks for the ground control experiments. The medium exchange and cell fixation at the experimental endpoint were automated following a preset schedule [25].

### 4.2. CellBox-1 Spaceflight Experiment

**Figure 7:**
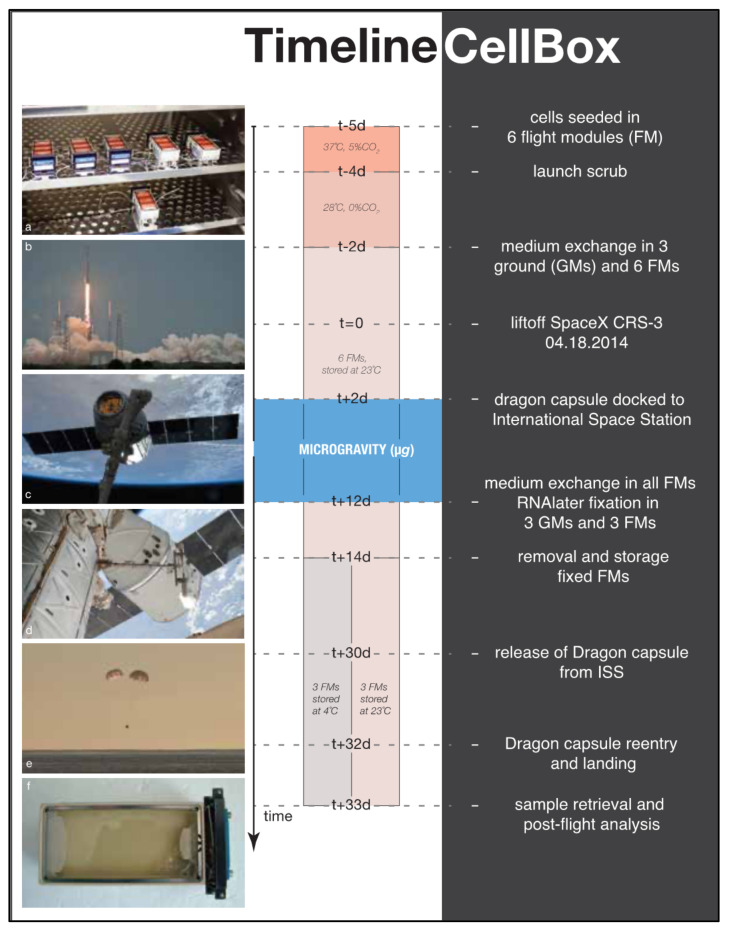
Timeline CellBox-1 experiment. (**a**) 1x10^6^ FTC-133 thyroid cancer cells seeded in six flight modules (FMs) incubated at 37 °C, 5%CO_2_. (**b**) Falcon-9 launches within Cargo Resupply Services mission 3 (SpaceX CRS-3). (**c**) Dragon capsule adjacent to International Space Station (ISS). (**d**) SpaceX capsule docked to ISS. (**e**) Capsule reentry and Pacific Ocean landing. (**f**) Flight module inspection before post-flight analysis (picture a courtesy RaisFoto, pictures b-e photo credit SpaceX).

The CellBox-1 spaceflight experiment was conducted as part of the SpaceX CRS-3 Commercial Resupply Service mission, launched on 18 April 2014 (https://www.dlr.de/content/de/bilder/2014/2/start-des-des-cellbox-experiments_15157.html), Riwaldt et al. reported methods and results in detail [25]. In short, 1 × 10^6^ FTC-133 were seeded in nine cell containers (FM) and incubated at 37 °C and 5% CO_2_. Twenty-four hours prior to launch, three FMs were transferred to an incubation chamber at 23 °C at Cape Canaveral to serve as ground controls (GM). The remaining six FMs were moved to the Dragon capsule and flown to the International Space Station (ISS). Upon arrival at the ISS, the FMs were incubated continuously at 23 °C at a *g*-force oscillating around ±0.005 *g.* After 12 days of microgravity, the cells of three FMs were fixed using RNA*later* via an automated process [24], collecting the aspirated media. The remaining three FMs were continuously incubated at 23 °C for the remainder of the spaceflight and, therefore, were not included in this analysis. The GMs were treated identically and simultaneously on the ground. Forty-eight hours post-fixation, both the FMs and GMs were cooled to 4 °C. After the return of the Dragon vessel on 20 May 2014, all containers were returned to the lab for cell and cell supernatant harvest (Figure 7).

### 4.3. Exosome Harvest and Isolation

Following harvest, the cell supernatants of the FMs and GMs underwent an adjusted differential centrifugation protocol [149]. Two consecutive spins at 300× *g* (10 min, 4 °C) using a swinging bucket rotor, followed by centrifugation at 2500× *g* (15 min, 4 °C, twice) pelleted cells, cell debris, and large vesicles. Additional high-speed centrifugation was not necessary as the analysis via ExoView^®^ does not require preceding particle isolation. The collected supernatants were divided into 2 mL aliquots and stored at −80 °C until further analysis.

### 4.4. TaqMan™ Advanced miRNA Arrays

As previously reported, we have analyzed the CellBox-1 samples regarding their exosome number, size distribution and tetraspanin colocalization pattern via ExoView using the EV-TETRA-C ExoView Tetraspanin Kit (NanoView Biosciences, Boston, MA, USA) [63]. One sample each was used for further analysis of the miRNA content and to scan for expression changes. The method of choice was the TaqMan™ Advanced miRNA Array, using the Human A and B Cards (ThermoFisher Scientific, Waltham, MA, USA) according to the manufacturer’s protocol. In summary, following the exosome harvest, the total RNA cargo was isolated, and the miRNA present was transcribed into cDNA via a TaqMan™ Advanced miRNA cDNA Synthesis Kit (ThermoFisher Scientific, Waltham, MA, USA). The resulting cDNA template was diluted 1:10 and mixed with the remaining reaction components (TaqMan^®^ Fast Advanced Master Mix (2x), ThermoFisher Scientific, Waltham, MA, USA, RNase-free water) and applied onto the array cards. The sealed cards were loaded into the 7900HT Fast Real-Time PCR System with 384-Well Block Module (ThermoFisher Scientific, Waltham, MA, USA) and amplified with the following thermal protocol (Table 2).

**Table 2:** TaqMan™ Advanced miRNA Array Thermal Protocol.

Step	Temperature	Time	Cycles
Enzyme activation	92 °C	10 min	1
Denature	95 °C	1 s	40
Anneal/Extend	60 °C	20 s	

The analysis of the results was carried out with ThermoFisher’s ExpressionSuite^TM^ Software and displayed as a Comparative C_t_ (ΔΔC_t_).

### 4.5. Exosome Isolation by Size Exclusion Chromatography

The samples were centrifuged at 300× *g* for 10 min, followed by centrifugation at 3000× *g* for 10 min. The clarified supernatant was then concentrated to approximately 500 μL on a 100 KD Amicon Ultra Centrifugal filter (Millipore, Temecula, CA, USA). The exosomes were then isolated from the concentrated supernatant by size exclusion chromatography (SEC). Sephacryl S-300 High Resolution (GE Healthcare, Chicago, IL, USA) was packed on a glass econo-column chromatography column (BioRad, Hercules, CA, USA) (10 cm height, 1.5 cm diameter). The column was washed with 0.32% Sodium Citrate in PBS, and the supernatant was loaded onto the column and allowed to enter the resin by gravity flow. The eluate was collected in 23 fractions of 15 drops (~500 μL) on a Model 2110 Fraction Collector (BioRad, Hercules, CA, USA). For each fraction, the presence of exosomes was determined by nanoparticle tracking analysis as described below. The exosome-containing fractions were then further concentrated to 1/100th of the original supernatant.

### 4.6. Exosome Characterization by Nanoparticle Tracking Analysis

The isolated exosomes were analyzed with a NanoSight NS300 (Malvern Panalytical Inc., Westborough, MA, USA). The NS300 allows rapid analysis of the size distribution and concentration of all types of nanoparticles from 0.01 to 1 µm in diameter. Particles in liquid suspension were loaded into a sample chamber, which is illuminated by a laser beam. Particles in the path of the beam scatter the laser light and is viewed with a digital camera, which captures a video of the particles moving under Brownian motion. The Nanoparticle Tracking Analysis (NTA) software analyses the particles individually and simultaneously (particle-by-particle), and by using the Stokes Einstein equation, calculates their hydrodynamic diameters [150].

### 4.7. TaqMan™ Advanced Single-Tube miRNA Assays

The validation of our selected miRNA target followed the protocol described above in Section 4.4., differing in the use of single-tube assays for hsa-miR-429, hsa-miR-128-3p, and hsa-miR-199a-5p rather than array cards and the use of C. elegans miR-39 as a normalizer (C39). Exosomes, and subsequently the contained RNA, were isolated from the remaining FM and GM as described above. After transcription of the received miRNA to cDNA (TaqMan™ Advanced miRNA cDNA Synthesis Kit, ThermoFisher Scientific, Waltham, MA, USA), the targets were quantitatively amplified using the thermal protocol below (Table 3) using a CFX96 Touch Real-Time PCR System and analyzed with the CFX Maestro Software (Bio-Rad Laboratories, Inc., Hercules, CA, USA).

## 5. Conclusions

Our study results emphasize that exosomes and exosomal miRNAs can be critical tools to tie together the knowledge the microgravity research community has gathered so far. Even though this report focuses solely on thyroid cancers, the importance of the roles of exosomes and miRNAs in understanding and validating phenomena previously described is eminent. The use of cutting-edge technologies enabled us to further describe the cellular changes space travelers are subjected to, even years after the actual mission. Additionally, these analyses once again show how adaptable tumor cells react to even the slightest changes in the surrounding environment, be it microgravity or other related stressors. Further studies will certainly unravel and solidify necessary links on the path to finding a cure for cancer.

## Figures and Tables

**Table 3:** TaqMan™ Advanced *Single-tube miRNA Assay* Thermal Protocol.

Step	Temperature	Time	Cycles
Enzyme activation	95 °C	20 s	1
Denature	95 °C	3 s	40
Anneal/Extend	60 °C	30 s	

## Data Availability

Not applicable.

## Supplementary Materials

Supplementary materials can be found at https://www.mdpi.com/article/10.3390/ijms222312841/s1.

## Abbreviations

AB	Antibody
CTGF	Connective Tissue Growth Factor
C39	C. elegans miR-39
EMT	Epithelial to Mesenchymal Transition
ESCC	Esophageal Squamous-Cell Cancer
EV	Extracellular Vesicle
GM	Ground Module
FM	Flight Module
FTC	Follicular Thyroid Carcinoma
ILV	Intraluminal Vesicle
MVBs	Multivesicular Bodies
ITGA3	Integrin α3β1
MTC	Medullary Thyroid Carcinoma
NTA	Nanoparticle Tracking Analysis
ON	Overnight
PTC	Papillary Thyroid Cancer
RISC	RNA-induced Silencing Complex
ROCK1	Rho-associated Coiled Coil-containing Protein Kinase 1
RPM	Random Positioning Machine
RQ	Relative Quantification
RT	Room Temperature
r-μ*g*	Real Microgravity
s-μ*g*	Simulated Microgravity
SEC	Size Exclusion Chromatography
SD	Standard Deviation
SP-IRIS	Single-Particle Interferometric Reflectance Imaging Sensor
TAM	Tumor Associated Macrophage
TME	Tumor Microenvironment
TNBC	Triple-negative Breast Cancer
ZEB1	Zinc Finger E-box-binding Homeobox 1
