# Supplementary material for: Changes in Exosomal miRNA Composition in Thyroid Cancer Cells after Prolonged Exposure to Real Microgravity in Space
Source: Int J Mol Sci. 2021 Nov 27;22(23):12841. doi: 10.3390/ijms222312841 (PMC8657878; doi:10.3390/ijms222312841)
Supplement: Supplementary file 1 [file ijms-22-12841-s001.zip › ijms-1471759-supplementary.pdf]

NANOSIGHT

F1 F5 2021-09-16 14-08-03

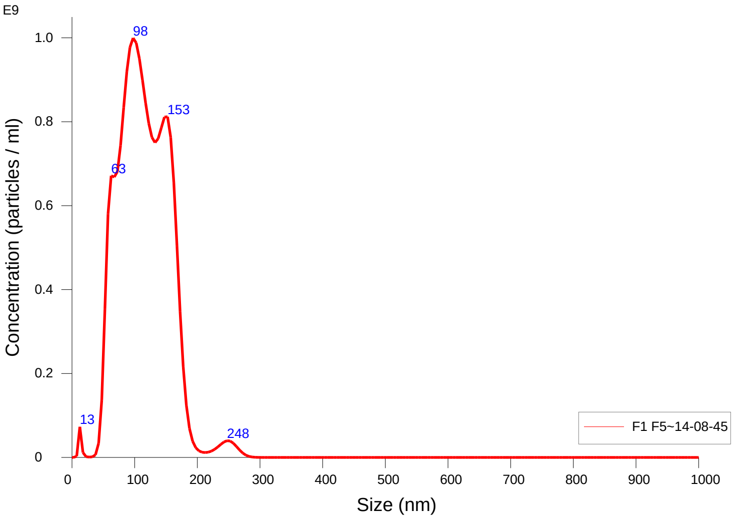

FTLA Concentration / Size graph for Experiment:
F1 F5 2021-09-16 14-08-03

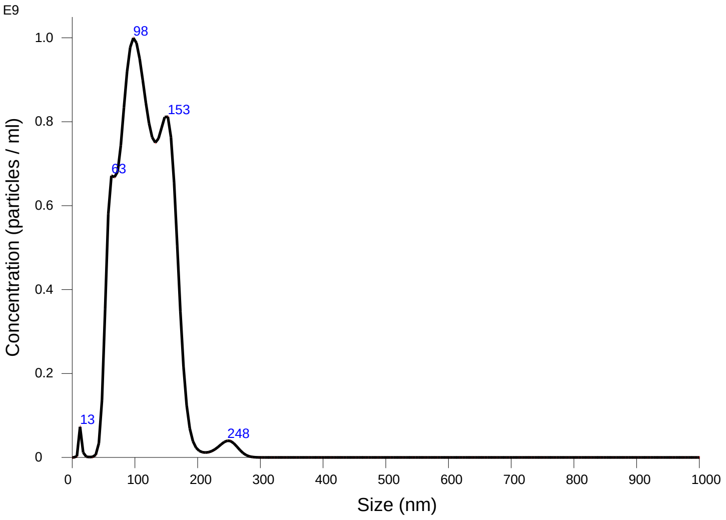

Averaged FTLA Concentration / Size for Experiment:
F1 F5 2021-09-16 14-08-03
Error bars indicate + / -1 standard error of the mean

Included Files

F1 F5 2021-09-16 14-08-45

Details

NTA Version:	NTA 3.4 Build 3.4.003
Script Used:	SOP Standard Measurement 02-08-03PM 16Sep2021.txt
Time Captured:	14:08:03 16/09/2021
Operator:	
Pre-treatment:	
Sample Name:	
Diluent:	
Remarks:	

Capture Settings

Camera Type:	sCMOS
Laser Type:	Green
Camera Level:	14
Slider Shutter:	1259
Slider Gain:	366
FPS	25.0
Number of Frames:	749
Temperature:	21.7 ºC
Viscosity:	(Water) 1.0 cP
Dilution factor:	1 x 10e2
Syringe Pump Speed:	25

Analysis Settings

Detect Threshold:	7
Blur Size:	Auto
Max Jump Distance:	Auto: 24.5 pix

Results

Stats: Merged Data

Mean:	114.9 nm
Mode:	98.3 nm
SD:	38.9 nm
D10:	65.9 nm
D50:	111.9 nm
D90:	162.6 nm

Stats: Mean +/- Standard Error

Mean:	114.9 +/- 0.0 nm
Mode:	98.3 +/- 0.0 nm
SD:	38.9 +/- 0.0 nm
D10:	65.9 +/- 0.0 nm
D50:	111.9 +/- 0.0 nm
D90:	162.6 +/- 0.0 nm
Concentration:	2.00e+10 +/- 0.00e+00 particles/ml
	10.9 +/- 0.0 particles/frame
	15.9 +/- 0.0 centres/frame

F1 F5 2021-09-16 14-08-03

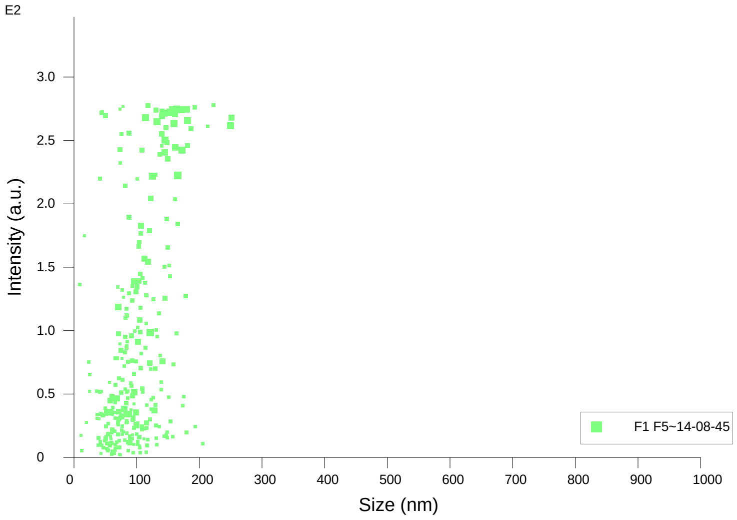

Intensity / Size graph for Experiment:

F1 F5 2021-09-16 14-08-03

NANOSIGHT

F1 F6 2021-09-16 14-02-43

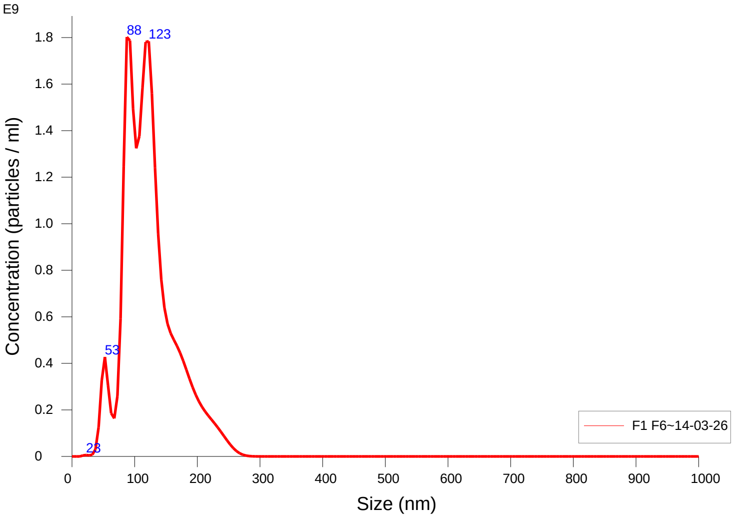

FTLA Concentration / Size graph for Experiment:
F1 F6 2021-09-16 14-02-43

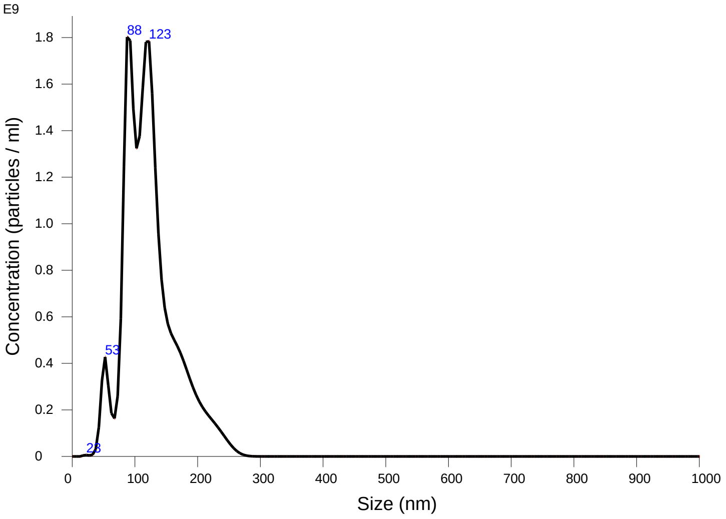

Averaged FTLA Concentration / Size for Experiment:
F1 F6 2021-09-16 14-02-43
Error bars indicate + / -1 standard error of the mean

Included Files

F1 F6 2021-09-16 14-03-26

Details

NTA Version:	NTA 3.4 Build 3.4.003
Script Used:	SOP Standard Measurement 02-02-43PM 16Sep2021.txt
Time Captured:	14:02:43 16/09/2021
Operator:	
Pre-treatment:	
Sample Name:	
Diluent:	
Remarks:	

Capture Settings

Camera Type:	sCMOS
Laser Type:	Green
Camera Level:	14
Slider Shutter:	1259
Slider Gain:	366
FPS	25.0
Number of Frames:	749
Temperature:	21.5 ºC
Viscosity:	(Water) 1.0 cP
Dilution factor:	1 x 10e2
Syringe Pump Speed:	25

Analysis Settings

Detect Threshold:	7
Blur Size:	Auto
Max Jump Distance:	Auto: 22.0 pix

Results

Stats: Merged Data

Mean:	123.0 nm
Mode:	89.9 nm
SD:	41.0 nm
D10:	81.3 nm
D50:	117.1 nm
D90:	182.0 nm

Stats: Mean +/- Standard Error

Mean:	123.0 +/- 0.0 nm
Mode:	89.9 +/- 0.0 nm
SD:	41.0 +/- 0.0 nm
D10:	81.3 +/- 0.0 nm
D50:	117.1 +/- 0.0 nm
D90:	182.0 +/- 0.0 nm
Concentration:	2.76e+10 +/- 0.00e+00 particles/ml
	15.1 +/- 0.0 particles/frame
	17.6 +/- 0.0 centres/frame

NANOSIGHT

F1 F6 2021-09-16 14-02-43

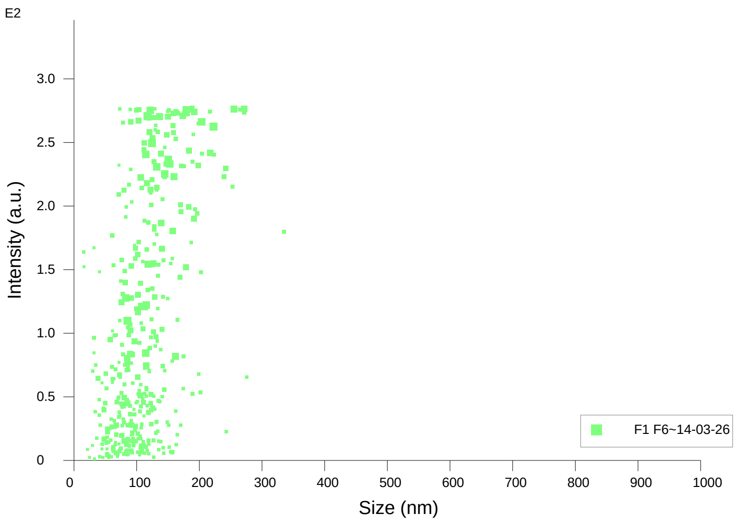

Intensity / Size graph for Experiment:
F1 F6 2021-09-16 14-02-43

NANOSIGHT

F2 F5 2021-09-16 14-49-47

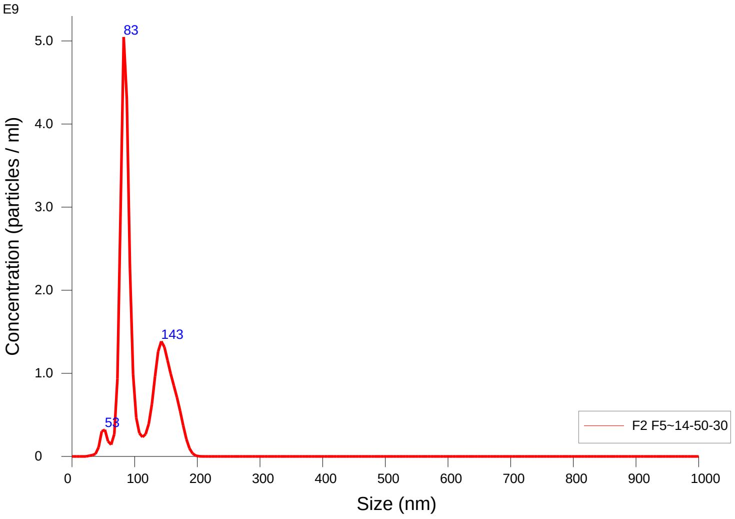

FTLA Concentration / Size graph for Experiment:
F2 F5 2021-09-16 14-49-47

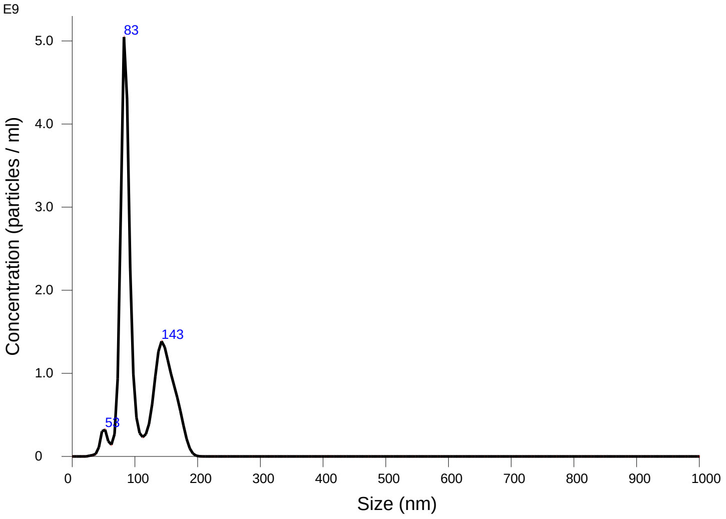

Averaged FTLA Concentration / Size for Experiment:
F2 F5 2021-09-16 14-49-47
Error bars indicate + / -1 standard error of the mean

Included Files

F2 F5 2021-09-16 14-50-30

Details

NTA Version:	NTA 3.4 Build 3.4.003
Script Used:	SOP Standard Measurement 02-49-47PM 16Sep2021.txt
Time Captured:	14:49:47 16/09/2021
Operator:	
Pre-treatment:	
Sample Name:	
Diluent:	
Remarks:	

Capture Settings

Camera Type:	sCMOS
Laser Type:	Green
Camera Level:	14
Slider Shutter:	1259
Slider Gain:	366
FPS	25.0
Number of Frames:	749
Temperature:	22.6 ºC
Viscosity:	(Water) 0.9 cP
Dilution factor:	1 x 10e2
Syringe Pump Speed:	25

Analysis Settings

Detect Threshold:	7
Blur Size:	Auto
Max Jump Distance:	Auto: 23.1 pix

Results

Stats: Merged Data

Mean:	107.9 nm
Mode:	83.7 nm
SD:	34.3 nm
D10:	76.2 nm
D50:	90.9 nm
D90:	159.1 nm

Stats: Mean +/- Standard Error

Mean:	107.9 +/- 0.0 nm
Mode:	83.7 +/- 0.0 nm
SD:	34.3 +/- 0.0 nm
D10:	76.2 +/- 0.0 nm
D50:	90.9 +/- 0.0 nm
D90:	159.1 +/- 0.0 nm
Concentration:	3.00e+10 +/- 0.00e+00 particles/ml
	16.4 +/- 0.0 particles/frame
	19.0 +/- 0.0 centres/frame

NANOSIGHT

F2 F5 2021-09-16 14-49-47

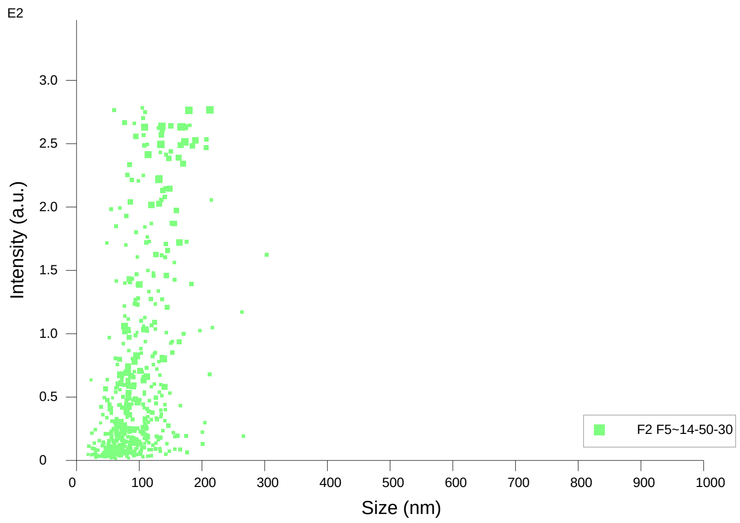

Intensity / Size graph for Experiment:
F2 F5 2021-09-16 14-49-47

NANOSIGHT

F2 F6 2021-09-16 14-46-27

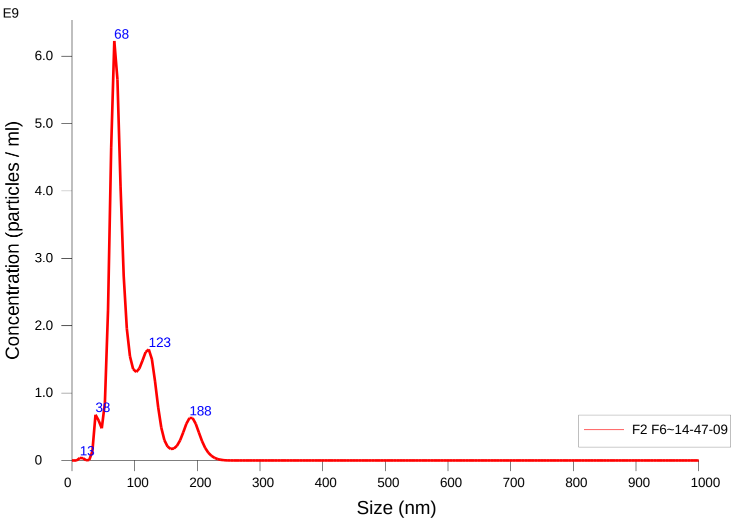

FTLA Concentration / Size graph for Experiment:
F2 F6 2021-09-16 14-46-27

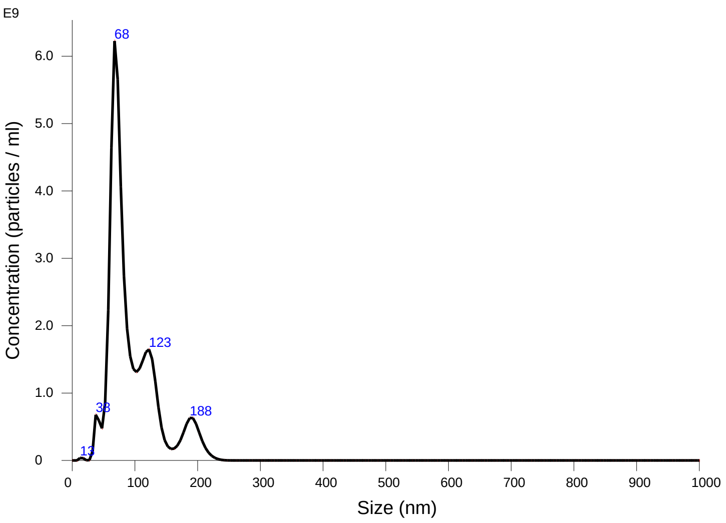

Averaged FTLA Concentration / Size for Experiment:
F2 F6 2021-09-16 14-46-27
Error bars indicate + / -1 standard error of the mean

Included Files

F2 F6 2021-09-16 14-47-09

Details

NTA Version:	NTA 3.4 Build 3.4.003
Script Used:	SOP Standard Measurement 02-46-27PM 16Sep2021.txt
Time Captured:	14:46:27 16/09/2021
Operator:	
Pre-treatment:	
Sample Name:	
Diluent:	
Remarks:	

Capture Settings

Camera Type:	sCMOS
Laser Type:	Green
Camera Level:	14
Slider Shutter:	1259
Slider Gain:	366
FPS	25.0
Number of Frames:	749
Temperature:	22.6 ^{o}C
Viscosity:	(Water) 0.9 cP
Dilution factor:	1 x 10e2
Syringe Pump Speed:	25

Analysis Settings

Detect Threshold:	7
Blur Size:	Auto
Max Jump Distance:	Auto: 23.2 pix

Results

Stats: Merged Data

Mean:	94.3 nm
Mode:	68.7 nm
SD:	39.7 nm
D10:	59.9 nm
D50:	79.1 nm
D90:	149.8 nm

Stats: Mean +/- Standard Error

Mean:	94.3 +/- 0.0 nm
Mode:	68.7 +/- 0.0 nm
SD:	39.7 +/- 0.0 nm
D10:	59.9 +/- 0.0 nm
D50:	79.1 +/- 0.0 nm
D90:	149.8 +/- 0.0 nm
Concentration:	4.99e+10 +/- 0.00e+00 particles/ml
	27.2 +/- 0.0 particles/frame
	31.2 +/- 0.0 centres/frame

F2 F6 2021-09-16 14-46-27

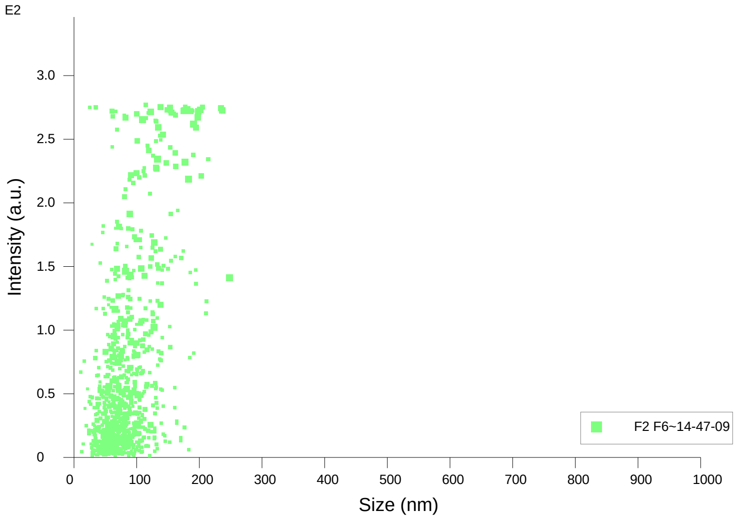

Intensity / Size graph for Experiment:
F2 F6 2021-09-16 14-46-27

NANOSIGHT

F3 F6 2021-06-22 10-30-49

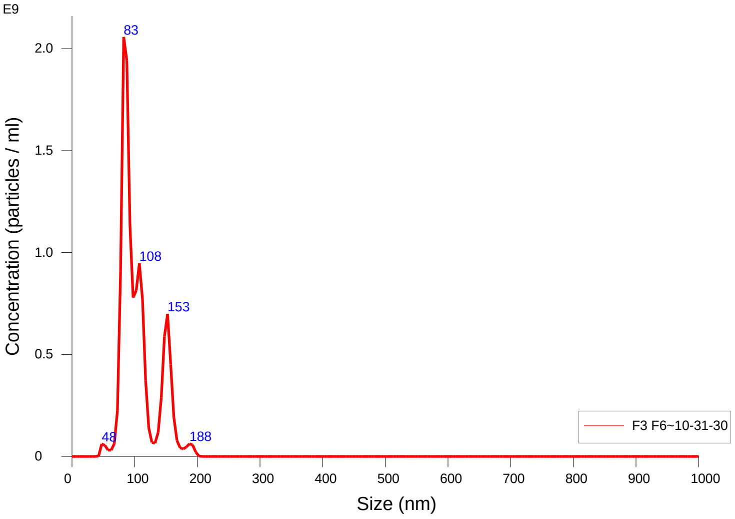

FTLA Concentration / Size graph for Experiment:
F3 F6 2021-06-22 10-30-49

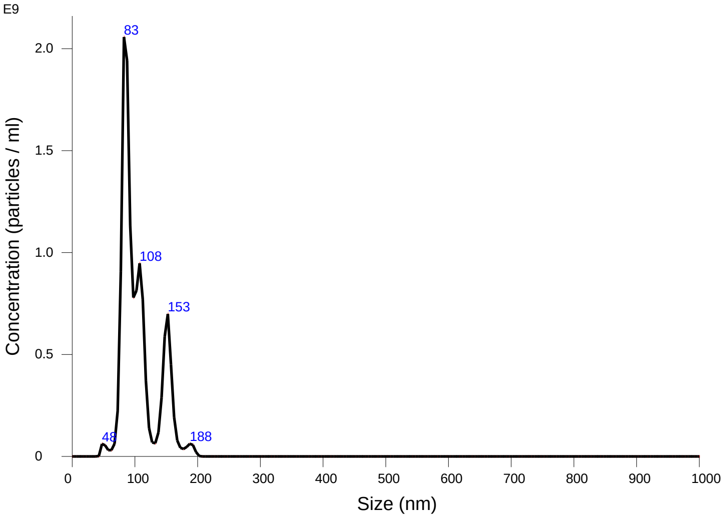

Averaged FTLA Concentration / Size for Experiment:
F3 F6 2021-06-22 10-30-49
Error bars indicate + / -1 standard error of the mean

Included Files

F3 F6 2021-06-22 10-31-30

Details

NTA Version:	NTA 3.4 Build 3.4.003
Script Used:	SOP Standard Measurement 10-30-49AM 22Jun2021.txt
Time Captured:	10:30:49 22/06/2021
Operator:	
Pre-treatment:	
Sample Name:	
Diluent:	
Remarks:	

Capture Settings

Camera Type:	sCMOS
Laser Type:	Green
Camera Level:	14
Slider Shutter:	1259
Slider Gain:	366
FPS	25.0
Number of Frames:	749
Temperature:	23.6 ^{o}C
Viscosity:	(Water) 0.9 cP
Dilution factor:	1 x 10e2
Syringe Pump Speed:	25

Analysis Settings

Detect Threshold:	7
Blur Size:	Auto
Max Jump Distance:	Auto: 14.2 pix

Results

Stats: Merged Data

Mean:	105.5 nm
Mode:	84.5 nm
SD:	28.5 nm
D10:	79.7 nm
D50:	95.4 nm
D90:	152.7 nm

Stats: Mean +/- Standard Error

Mean:	105.5 +/- 0.0 nm
Mode:	84.5 +/- 0.0 nm
SD:	28.5 +/- 0.0 nm
D10:	79.7 +/- 0.0 nm
D50:	95.4 +/- 0.0 nm
D90:	152.7 +/- 0.0 nm
Concentration:	1.31e+10 +/- 0.00e+00 particles/ml
	7.2 +/- 0.0 particles/frame
	9.1 +/- 0.0 centres/frame

NANOSIGHT

F3 F6 2021-06-22 10-30-49

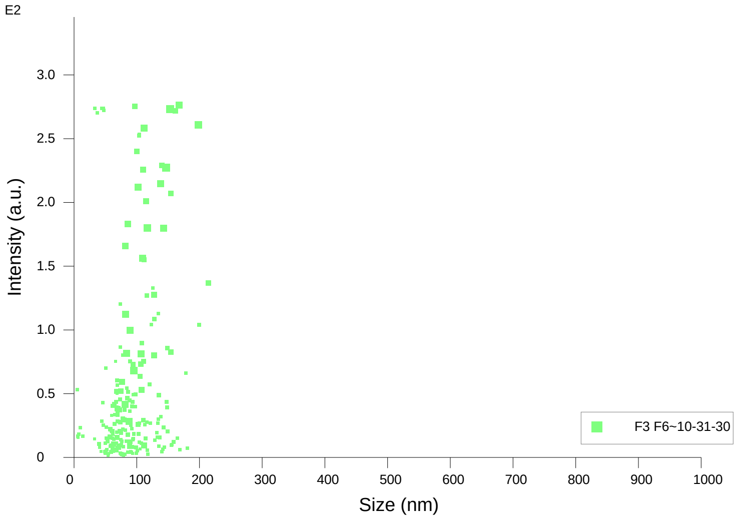

Intensity / Size graph for Experiment:
F3 F6 2021-06-22 10-30-49

NANOSIGHT

F3 F7 2021-06-22 09-55-20

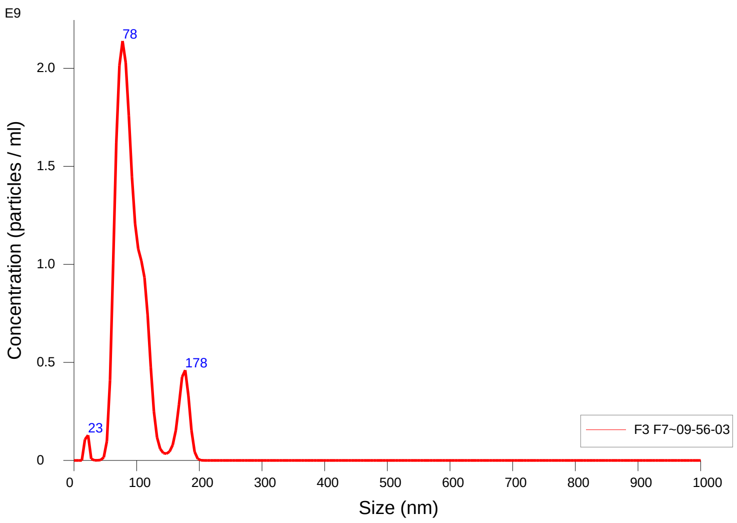

FTLA Concentration / Size graph for Experiment:
F3 F7 2021-06-22 09-55-20

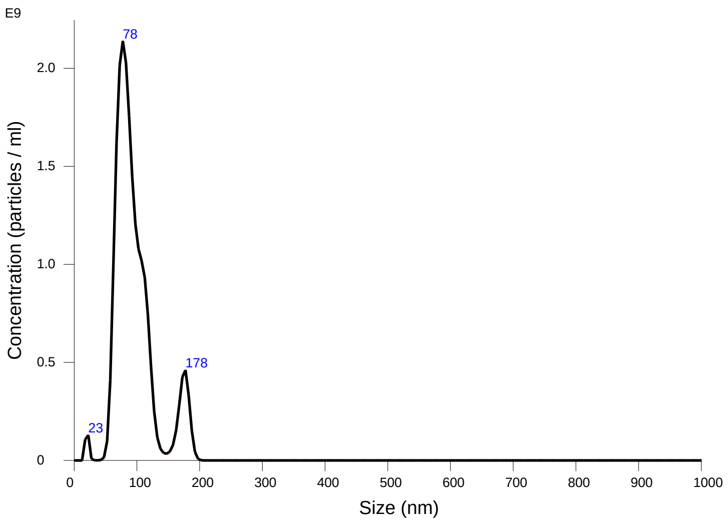

Averaged FTLA Concentration / Size for Experiment:
F3 F7 2021-06-22 09-55-20
Error bars indicate + / -1 standard error of the mean

Included Files

F3 F7 2021-06-22 09-56-03

Details

NTA Version:	NTA 3.4 Build 3.4.003
Script Used:	SOP Standard Measurement 09-55-20AM 22Jun2021.txt
Time Captured:	09:55:20 22/06/2021
Operator:	
Pre-treatment:	
Sample Name:	
Diluent:	
Remarks:	

Capture Settings

Camera Type:	sCMOS
Laser Type:	Green
Camera Level:	14
Slider Shutter:	1259
Slider Gain:	366
FPS	25.0
Number of Frames:	749
Temperature:	21.9 ºC
Viscosity:	(Water) 1.0 cP
Dilution factor:	1 x 10e2
Syringe Pump Speed:	25

Analysis Settings

Detect Threshold:	7
Blur Size:	Auto
Max Jump Distance:	Auto: 27.9 pix

Results

Stats: Merged Data

Mean:	95.3 nm
Mode:	77.7 nm
SD:	32.0 nm
D10:	65.9 nm
D50:	87.2 nm
D90:	138.7 nm

Stats: Mean +/- Standard Error

Mean:	95.3 +/- 0.0 nm
Mode:	77.7 +/- 0.0 nm
SD:	32.0 +/- 0.0 nm
D10:	65.9 +/- 0.0 nm
D50:	87.2 +/- 0.0 nm
D90:	138.7 +/- 0.0 nm
Concentration:	2.07e+10 +/- 0.00e+00 particles/ml
	11.3 +/- 0.0 particles/frame
	14.0 +/- 0.0 centres/frame

NANOSIGHT

F3 F7 2021-06-22 09-55-20

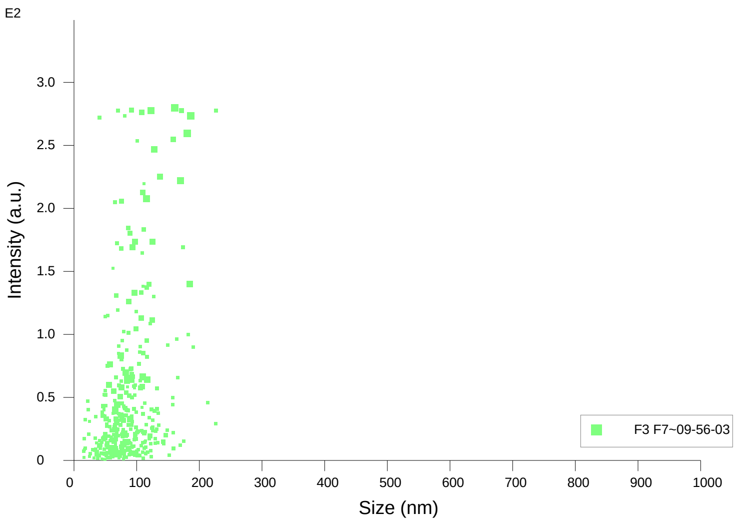

Intensity / Size graph for Experiment:
F3 F7 2021-06-22 09-55-20

NANOSIGHT

G1 F5 2021-09-16 14-30-52

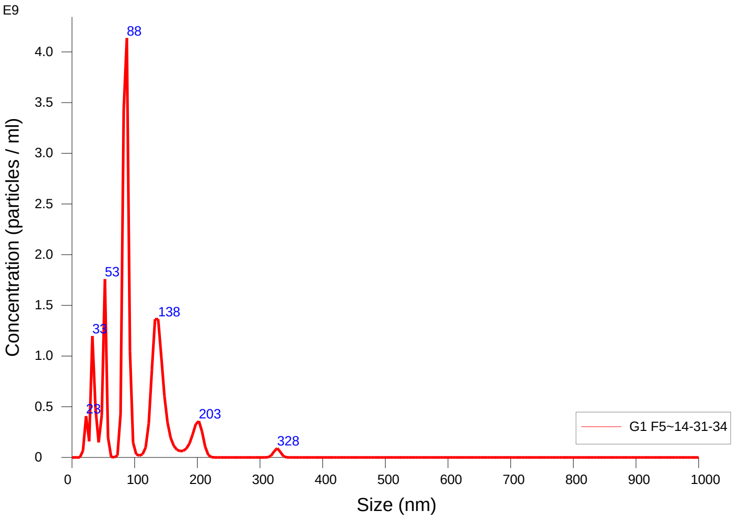

FTLA Concentration / Size graph for Experiment:
G1 F5 2021-09-16 14-30-52

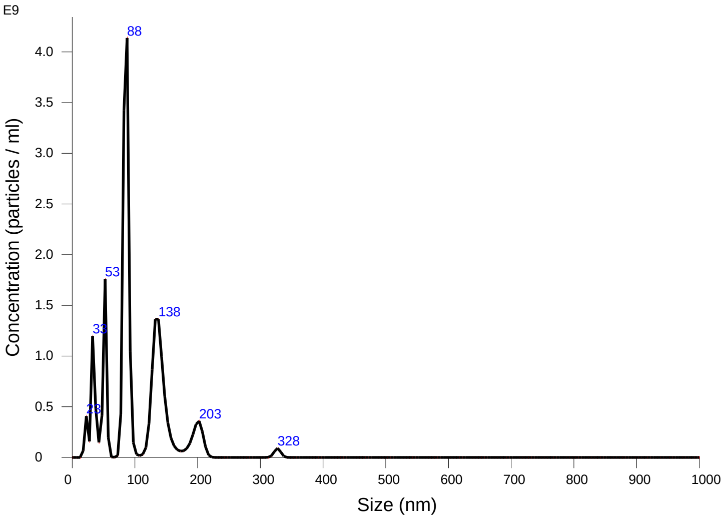

Averaged FTLA Concentration / Size for Experiment:
G1 F5 2021-09-16 14-30-52
Error bars indicate + / -1 standard error of the mean

Included Files

G1 F5 2021-09-16 14-31-34

Details

NTA Version:	NTA 3.4 Build 3.4.003
Script Used:	SOP Standard Measurement 02-30-52PM 16Sep2021.txt
Time Captured:	14:30:52 16/09/2021
Operator:	
Pre-treatment:	
Sample Name:	
Diluent:	
Remarks:	

Capture Settings

Camera Type:	sCMOS
Laser Type:	Green
Camera Level:	14
Slider Shutter:	1259
Slider Gain:	366
FPS	25.0
Number of Frames:	749
Temperature:	22.3 ºC
Viscosity:	(Water) 0.9 cP
Dilution factor:	1 x 10e2
Syringe Pump Speed:	25

Analysis Settings

Detect Threshold:	7
Blur Size:	Auto
Max Jump Distance:	Auto: 22.8 pix

Results

Stats: Merged Data

Mean:	101.9 nm
Mode:	85.9 nm
SD:	50.3 nm
D10:	39.2 nm
D50:	88.0 nm
D90:	155.9 nm

Stats: Mean +/- Standard Error

Mean:	101.9 +/- 0.0 nm
Mode:	85.9 +/- 0.0 nm
SD:	50.3 +/- 0.0 nm
D10:	39.2 +/- 0.0 nm
D50:	88.0 +/- 0.0 nm
D90:	155.9 +/- 0.0 nm
Concentration:	2.24e+10 +/- 0.00e+00 particles/ml
	12.2 +/- 0.0 particles/frame
	15.9 +/- 0.0 centres/frame

NANOSIGHT

G1 F5 2021-09-16 14-30-52

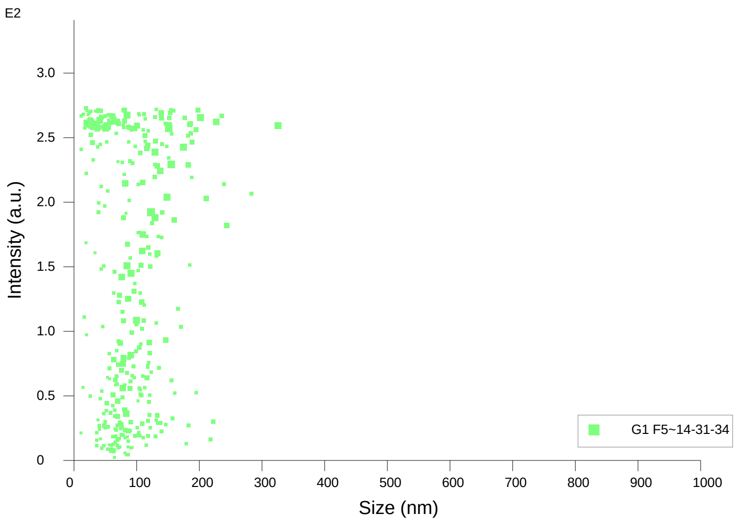

Intensity / Size graph for Experiment:
G1 F5 2021-09-16 14-30-52

NANOSIGHT

G1 F6 2021-09-16 14-22-18

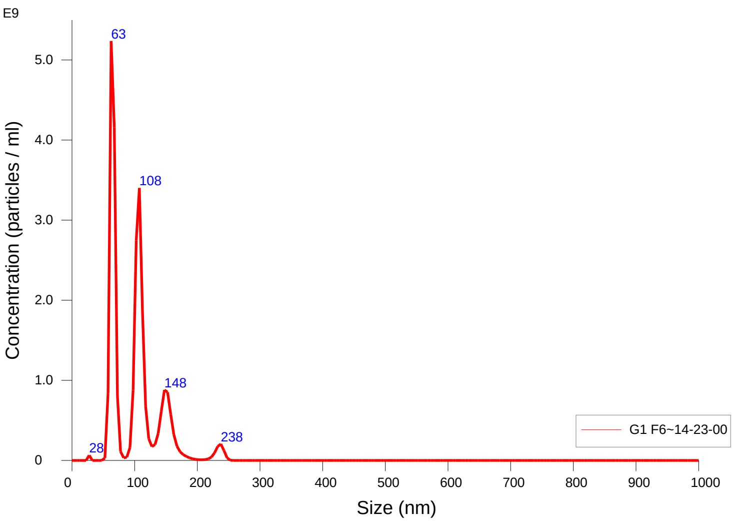

FTLA Concentration / Size graph for Experiment:
G1 F6 2021-09-16 14-22-18

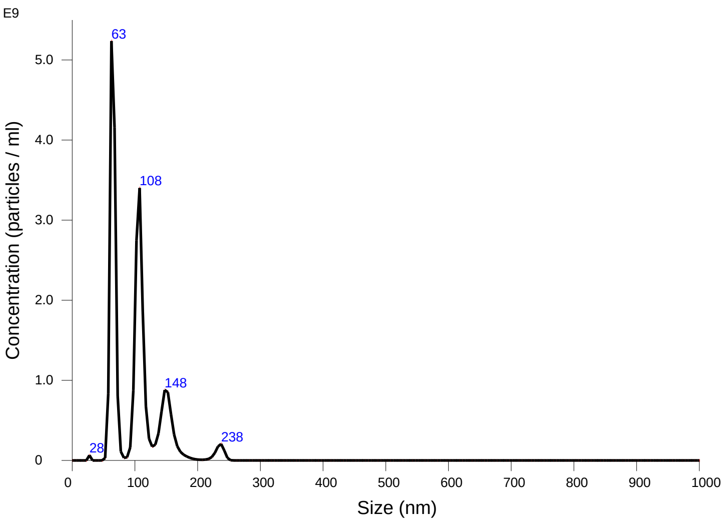

Averaged FTLA Concentration / Size for Experiment:
G1 F6 2021-09-16 14-22-18
Error bars indicate + / -1 standard error of the mean

Included Files

G1 F6 2021-09-16 14-23-00

Details

NTA Version:	NTA 3.4 Build 3.4.003
Script Used:	SOP Standard Measurement 02-22-18PM 16Sep2021.txt
Time Captured:	14:22:18 16/09/2021
Operator:	
Pre-treatment:	
Sample Name:	
Diluent:	
Remarks:	

Capture Settings

Camera Type:	sCMOS
Laser Type:	Green
Camera Level:	14
Slider Shutter:	1259
Slider Gain:	366
FPS	25.0
Number of Frames:	749
Temperature:	22.0 ºC
Viscosity:	(Water) 1.0 cP
Dilution factor:	1 x 10e2
Syringe Pump Speed:	25

Analysis Settings

Detect Threshold:	7
Blur Size:	Auto
Max Jump Distance:	Auto: 24.3 pix

Results

Stats: Merged Data

Mean:	99.8 nm
Mode:	64.0 nm
SD:	39.1 nm
D10:	61.6 nm
D50:	101.6 nm
D90:	151.8 nm

Stats: Mean +/- Standard Error

Mean:	99.8 +/- 0.0 nm
Mode:	64.0 +/- 0.0 nm
SD:	39.1 +/- 0.0 nm
D10:	61.6 +/- 0.0 nm
D50:	101.6 +/- 0.0 nm
D90:	151.8 +/- 0.0 nm
Concentration:	2.65e+10 +/- 0.00e+00 particles/ml
	14.5 +/- 0.0 particles/frame
	17.2 +/- 0.0 centres/frame

G1 F6 2021-09-16 14-22-18

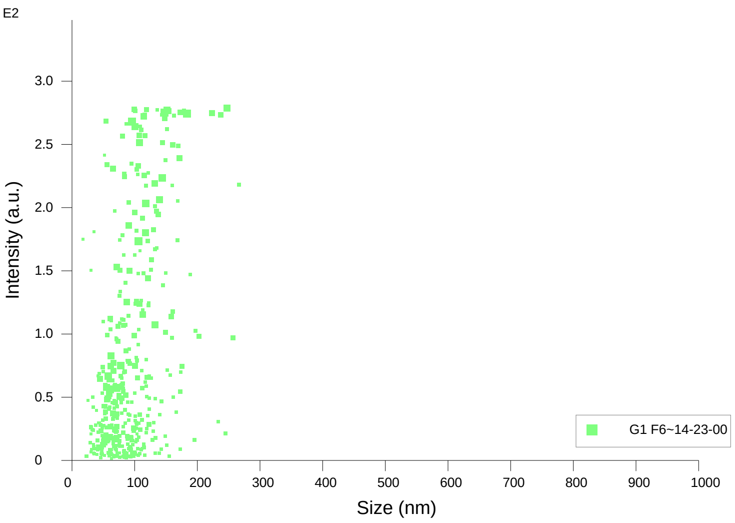

Intensity / Size graph for Experiment:

G1 F6 2021-09-16 14-22-18

NANOSIGHT

G2 F7 2021-09-16 14-55-50

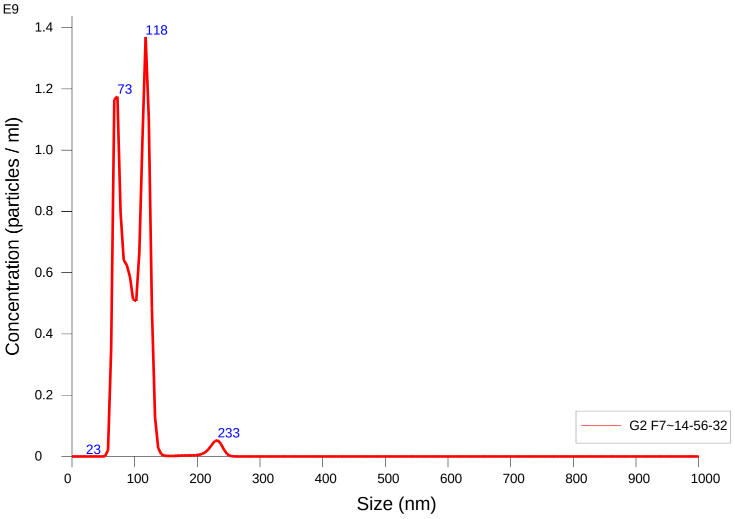

FTLA Concentration / Size graph for Experiment:
G2 F7 2021-09-16 14-55-50

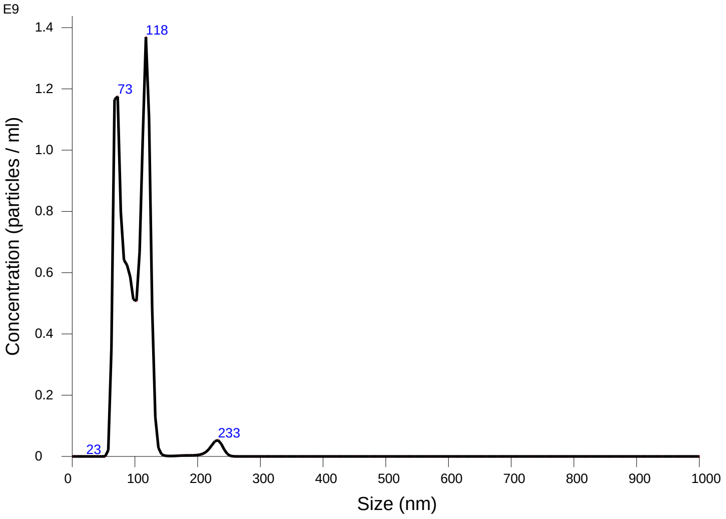

Averaged FTLA Concentration / Size for Experiment:
G2 F7 2021-09-16 14-55-50
Error bars indicate + / -1 standard error of the mean

Included Files

G2 F7 2021-09-16 14-56-32

Details

NTA Version:	NTA 3.4 Build 3.4.003
Script Used:	SOP Standard Measurement 02-55-50PM 16Sep2021.txt
Time Captured:	14:55:50 16/09/2021
Operator:	
Pre-treatment:	
Sample Name:	
Diluent:	
Remarks:	

Capture Settings

Camera Type:	sCMOS
Laser Type:	Green
Camera Level:	14
Slider Shutter:	1259
Slider Gain:	366
FPS	25.0
Number of Frames:	749
Temperature:	22.7 ºC
Viscosity:	(Water) 0.9 cP
Dilution factor:	1 x 10e2
Syringe Pump Speed:	25

Analysis Settings

Detect Threshold:	7
Blur Size:	Auto
Max Jump Distance:	Auto: 14.2 pix

Results

Stats: Merged Data

Mean:	99.6 nm
Mode:	117.8 nm
SD:	29.0 nm
D10:	68.3 nm
D50:	98.8 nm
D90:	124.0 nm

Stats: Mean +/- Standard Error

Mean:	99.6 +/- 0.0 nm
Mode:	117.8 +/- 0.0 nm
SD:	29.0 +/- 0.0 nm
D10:	68.3 +/- 0.0 nm
D50:	98.8 +/- 0.0 nm
D90:	124.0 +/- 0.0 nm
Concentration:	1.15e+10 +/- 0.00e+00 particles/ml
	6.3 +/- 0.0 particles/frame
	8.0 +/- 0.0 centres/frame

NANOSIGHT

G2 F7 2021-09-16 14-55-50

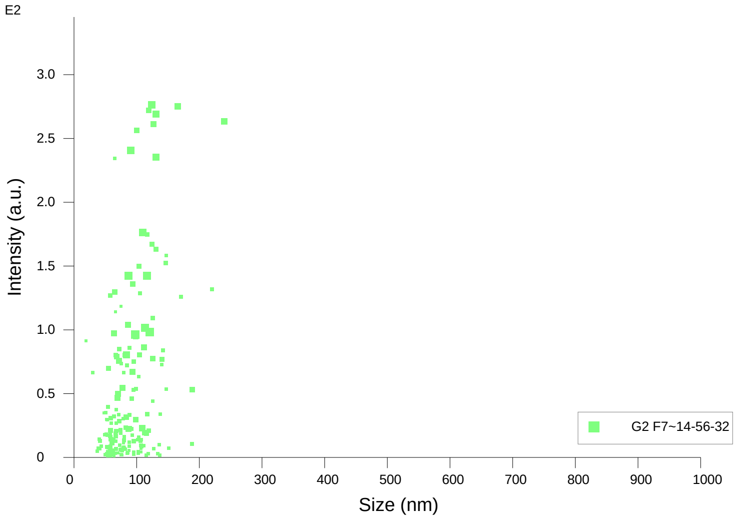

Intensity / Size graph for Experiment:
G2 F7 2021-09-16 14-55-50

NANOSIGHT

G2 F8 2021-09-16 14-59-27

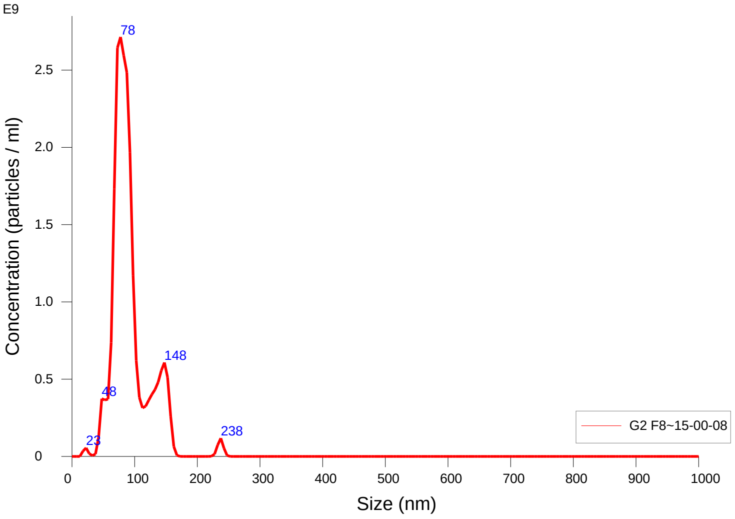

FTLA Concentration / Size graph for Experiment:
G2 F8 2021-09-16 14-59-27

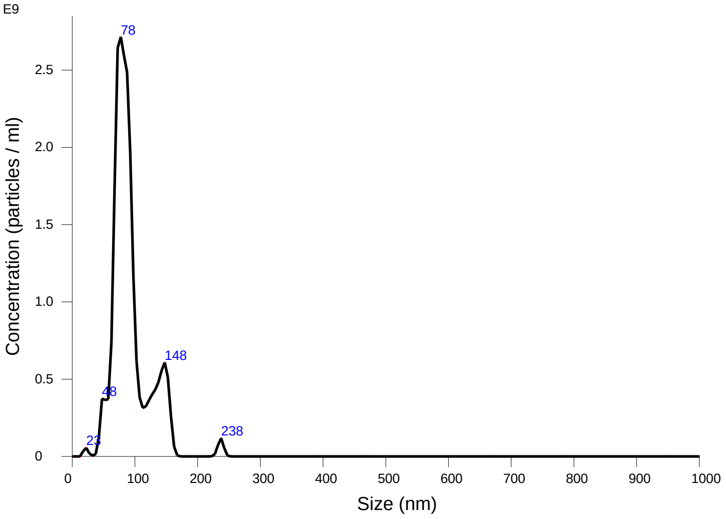

Averaged FTLA Concentration / Size for Experiment:
G2 F8 2021-09-16 14-59-27
Error bars indicate + / -1 standard error of the mean

Included Files

G2 F8 2021-09-16 15-00-08

Details

NTA Version:	NTA 3.4 Build 3.4.003
Script Used:	SOP Standard Measurement 02-59-27PM 16Sep2021.txt
Time Captured:	14:59:27 16/09/2021
Operator:	
Pre-treatment:	
Sample Name:	
Diluent:	
Remarks:	

Capture Settings

Camera Type:	sCMOS
Laser Type:	Green
Camera Level:	14
Slider Shutter:	1259
Slider Gain:	366
FPS	25.0
Number of Frames:	749
Temperature:	22.7 ºC
Viscosity:	(Water) 0.9 cP
Dilution factor:	1 x 10e2
Syringe Pump Speed:	25

Analysis Settings

Detect Threshold:	7
Blur Size:	Auto
Max Jump Distance:	Auto: 25.5 pix

Results

Stats: Merged Data

Mean:	92.1 nm
Mode:	76.9 nm
SD:	30.6 nm
D10:	65.6 nm
D50:	84.5 nm
D90:	139.7 nm

Stats: Mean +/- Standard Error

Mean:	92.1 +/- 0.0 nm
Mode:	76.9 +/- 0.0 nm
SD:	30.6 +/- 0.0 nm
D10:	65.6 +/- 0.0 nm
D50:	84.5 +/- 0.0 nm
D90:	139.7 +/- 0.0 nm
Concentration:	2.30e+10 +/- 0.00e+00 particles/ml
	12.6 +/- 0.0 particles/frame
	14.7 +/- 0.0 centres/frame

G2 F8 2021-09-16 14-59-27

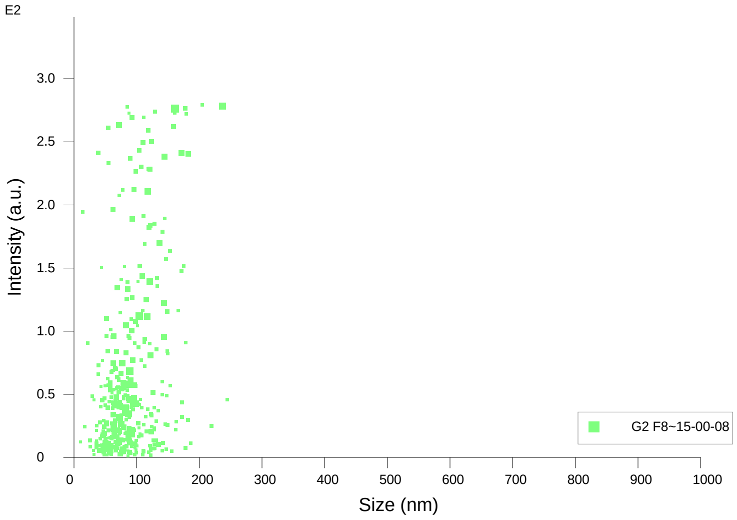

Intensity / Size graph for Experiment:

G2 F8 2021-09-16 14-59-27

NANOSIGHT

G3 F6 2021-06-22 10-23-15

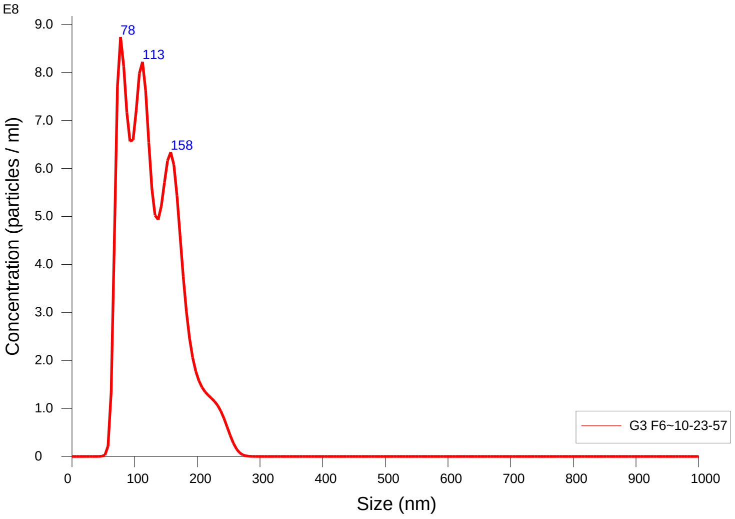

FTLA Concentration / Size graph for Experiment:
G3 F6 2021-06-22 10-23-15

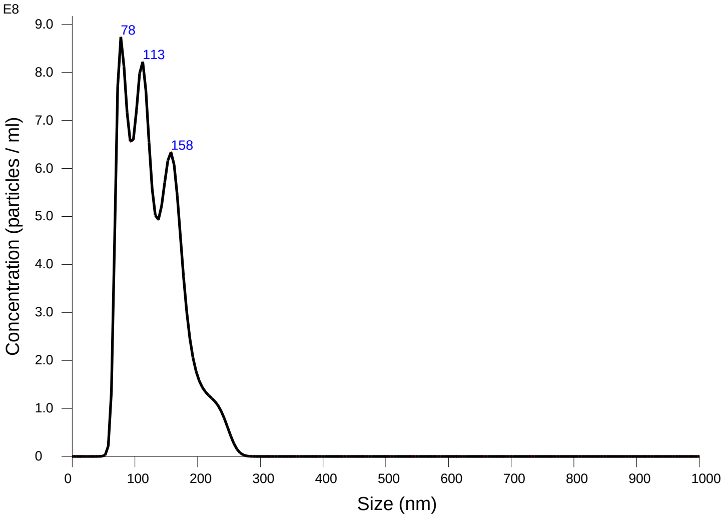

Averaged FTLA Concentration / Size for Experiment:
G3 F6 2021-06-22 10-23-15
Error bars indicate + / -1 standard error of the mean

Included Files

G3 F6 2021-06-22 10-23-57

Details

NTA Version:	NTA 3.4 Build 3.4.003
Script Used:	SOP Standard Measurement 10-23-15AM 22Jun2021.txt
Time Captured:	10:23:15 22/06/2021
Operator:	
Pre-treatment:	
Sample Name:	
Diluent:	
Remarks:	

Capture Settings

Camera Type:	sCMOS
Laser Type:	Green
Camera Level:	14
Slider Shutter:	1259
Slider Gain:	366
FPS	25.0
Number of Frames:	749
Temperature:	23.3 ºC
Viscosity:	(Water) 0.9 cP
Dilution factor:	1 x 10e2
Syringe Pump Speed:	25

Analysis Settings

Detect Threshold:	7
Blur Size:	Auto
Max Jump Distance:	Auto: 15.2 pix

Results

Stats: Merged Data

Mean:	129.4 nm
Mode:	78.2 nm
SD:	43.8 nm
D10:	76.8 nm
D50:	122.0 nm
D90:	188.7 nm

Stats: Mean +/- Standard Error

Mean:	129.4 +/- 0.0 nm
Mode:	78.2 +/- 0.0 nm
SD:	43.8 +/- 0.0 nm
D10:	76.8 +/- 0.0 nm
D50:	122.0 +/- 0.0 nm
D90:	188.7 +/- 0.0 nm
Concentration:	1.69e+10 +/- 0.00e+00 particles/ml
	9.2 +/- 0.0 particles/frame
	10.1 +/- 0.0 centres/frame

G3 F6 2021-06-22 10-23-15

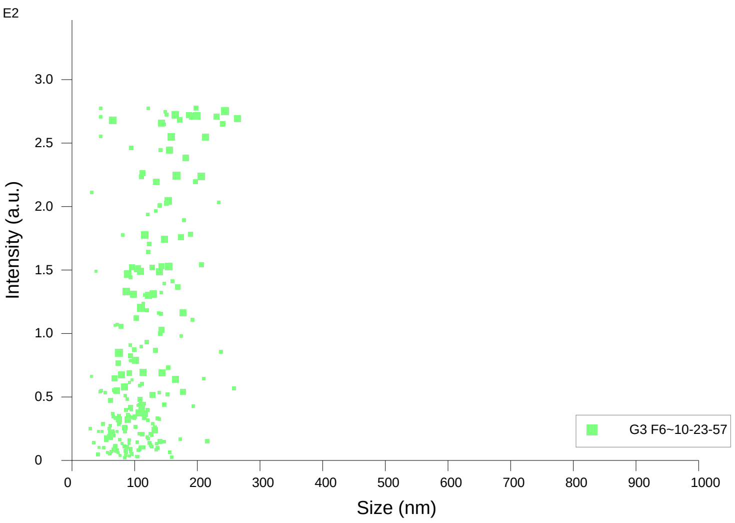

Intensity / Size graph for Experiment:
G3 F6 2021-06-22 10-23-15

NANOSIGHT

G3 F7 2021-06-22 10-09-10

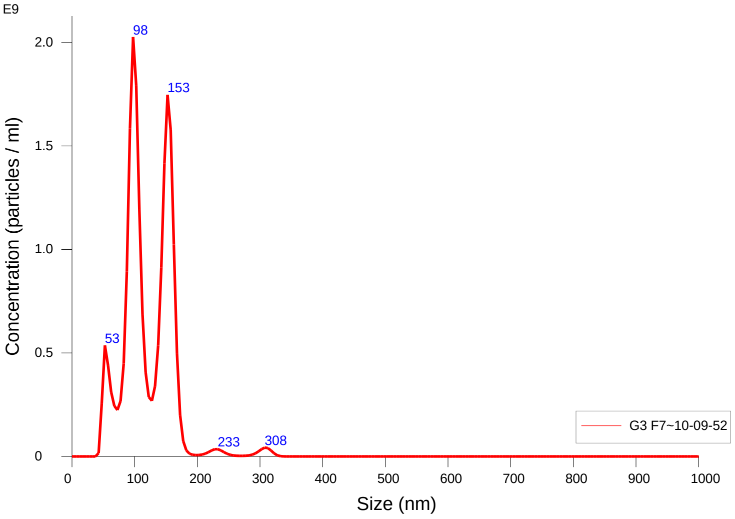

FTLA Concentration / Size graph for Experiment:
G3 F7 2021-06-22 10-09-10

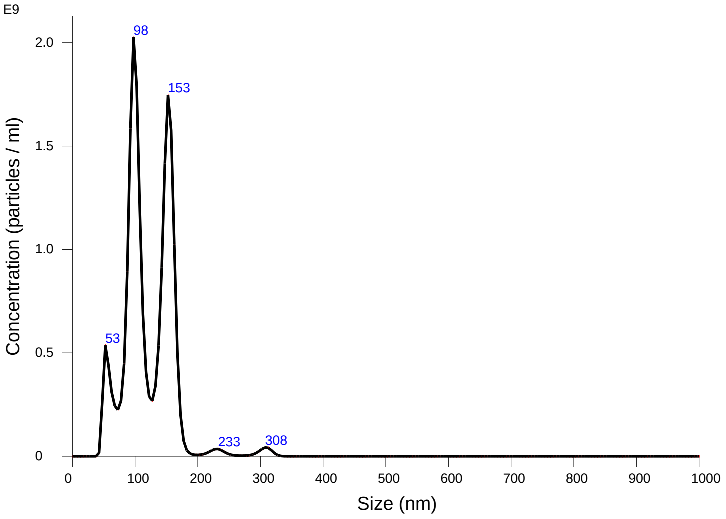

Averaged FTLA Concentration / Size for Experiment:
G3 F7 2021-06-22 10-09-10
Error bars indicate + / -1 standard error of the mean

Included Files

G3 F7 2021-06-22 10-09-52

Details

NTA Version:	NTA 3.4 Build 3.4.003
Script Used:	SOP Standard Measurement 10-09-10AM 22Jun2021.txt
Time Captured:	10:09:10 22/06/2021
Operator:	
Pre-treatment:	
Sample Name:	
Diluent:	
Remarks:	

Capture Settings

Camera Type:	sCMOS
Laser Type:	Green
Camera Level:	14
Slider Shutter:	1259
Slider Gain:	366
FPS	25.0
Number of Frames:	749
Temperature:	22.8 ºC
Viscosity:	(Water) 0.9 cP
Dilution factor:	1 x 10e2
Syringe Pump Speed:	25

Analysis Settings

Detect Threshold:	7
Blur Size:	Auto
Max Jump Distance:	Auto: 21.5 pix

Results

Stats: Merged Data

Mean:	121.0 nm
Mode:	98.3 nm
SD:	40.2 nm
D10:	75.8 nm
D50:	110.9 nm
D90:	161.2 nm

Stats: Mean +/- Standard Error

Mean:	121.0 +/- 0.0 nm
Mode:	98.3 +/- 0.0 nm
SD:	40.2 +/- 0.0 nm
D10:	75.8 +/- 0.0 nm
D50:	110.9 +/- 0.0 nm
D90:	161.2 +/- 0.0 nm
Concentration:	2.07e+10 +/- 0.00e+00 particles/ml
	11.3 +/- 0.0 particles/frame
	12.3 +/- 0.0 centres/frame

G3 F7 2021-06-22 10-09-10

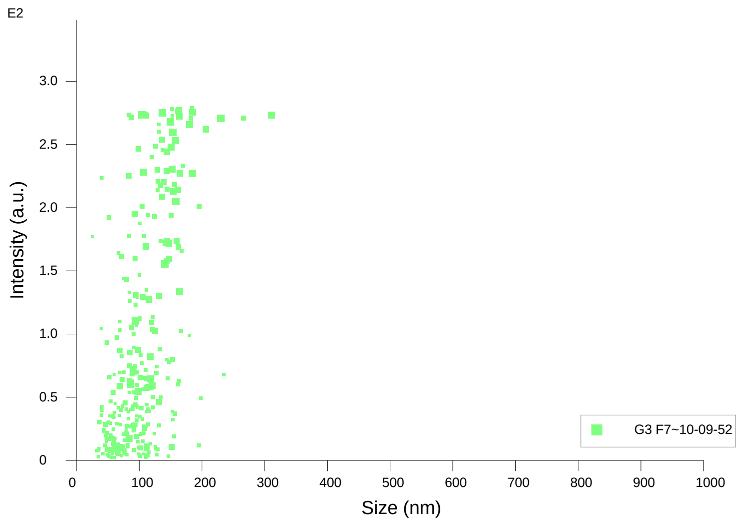

Intensity / Size graph for Experiment:
G3 F7 2021-06-22 10-09-10
